# Isolation and biological activities of compounds from *Rumex vesicarius* L. and their use as a component of a synbiotic preparation

**DOI:** 10.1016/j.fochx.2022.100306

**Published:** 2022-04-09

**Authors:** Ahmed Elbermawi, Mohamed Samir Darwish, Asmaa A. El-Awady, Ahmed A. Zaki, Longxin Qiu, Reham M. Samra

**Affiliations:** a Department of Pharmacognosy, Faculty of Pharmacy, Mansoura University, Mansoura, 35516 Egypt; b Dairy Department, Faculty of Agriculture, Mansoura University, Mansoura 35516, Egypt; c Department of Pharmacognosy, Faculty of Pharmacy, Horus University-Egypt, New Damietta 34518, Egypt; d Key Laboratory of Preventive Veterinary Medicine and Biotechnology, Longyan University, Longyan, 364012, P.R. China; e Fujian Provincial Key Laboratory for the Prevention and Control of Animal Infectious Diseases and Biotechnology, Longyan, 364012, P.R. China

**Keywords:** Ruby dock, Prebiotic, α- Amylase, Angiotensin-converting enzyme, *Escherichia coli* Nissle 1917, Protease, ACE, Angiotensin-converting enzyme, CFU, Colony forming units, EcN, *Escherichia coli*Nissle 1917, Lag, Lag time, NB, Nutrient broth, PI, Prebiotic index, P_score_, Prebiotic score, T_d_, Doubling time, Y_max_, Maximum growth at the stationary phase, µ_max_, specific growth rate

## Abstract

The study evaluated prebiotic potential and the enzyme inhibition of extracts and isolated compounds of *Rumex vesicarius* (ruby dock), family Polygonaceae. Eight known compounds were identified in the roots of *R. vesicarius*. Extracts and compounds (**1**–**8**) increased the growth rate of *Escherichia coli* Nissle 1917 differentially compared to controls. The highest prebiotic index (PI) and activity score was recorded for EcN in the presence of compound **4**, followed by, in descending order, petroleum ether, ethyl acetate, and total methanol extracts. The compounds and extracts reduced protease, α-amylase, and angiotensin-converting enzyme activities. This inhibitory activity was positively correlated with PI, P_score_, µ_u_, and Y_max_. These findings suggest that *R. vesicarius* is a good source of potential prebiotic and can boost beneficial bacteria. It may also be considered promising for treatment of diabetes mellitus, controlling weight, and regulating blood pressure.

## Highlights

• The most important points in our manuscript:
• Eight compounds; five phenolic, two sterols, and one sugar derivative were identified from the roots of *R. vesicarius*.
• All compounds and extracts promoted the growth of *E. coli* Nissle 1917 (EcN).
• The highest prebiotic index and activity score was recorded for EcN in the presence of the sugar derivative.
• The tested compounds and different extracts reduced the protease, α- amylase, and ACE activities.
• Correlation analysis demonstrated that the inhibitory activity against the tested enzymes is positively correlated with PI, P_score_, µ_u_, and Y_max_.

## 1 Introduction

Cardiovascular disorders and diabetes are considered noncommunicable diseases responsible for about 48 and 3.5 % of deaths worldwide in 2012 (Bello-Ovosi et al., 2018). Estimates of type 2 diabetes and hypertension were around 15.6% and 26.3%, respectively, for Egyptian adults (Hegazi et al., 2015, Ibrahim et al., 1995). Hypertension occurs in over two-thirds of type 2 diabetes patients, and coincides with the development of hyperglycemia. Increased hypertension may reflect pathological mechanisms, such as insulin resistance in the nitric-oxide pathway; the excitatory influences of hyperinsulinemia on sodium–fluid retention, smooth muscle growth, and sympathetic drive. Further, the stimulatory impacts of hyperglycemia on the renin-angiotensin system appears possible (Ferrannini & Cushman, 2012). High blood pressure leads to an increased risk of cardiovascular disease. Blood pressure lower than 140/85 mm Hg is a plausible therapeutic target in type 2 diabetes patients based on evidence from clinical trials. People with controlled type 2 diabetes have a similar risk of cardiovascular disease as hypertensive patients without diabetes (Ferrannini & Cushman, 2012). Antihypertensive and antidiabetic properties are found in medicinal plants worldwide. These natural remedies are used manage hypertension and diabetes concomitantly (Chukwuma et al., 2019).

*R. vesicarius* L. (family Polygonaceae), is the most common of nine recognized *Rumex* species (Laouini & Ouahrani, 2017). The plant is also known as bladder dock, ruby dock, and hummayd. The plant is edible and endemic to northeastern regions of Egypt. Both tender stems and leaves are consumed in North Africa and other parts of the world (Alfawaz, 2006, Laouini and Ouahrani, 2017). *R. vesicarius* is eaten fresh as sorrel and can be cooked (Al-Qura’n, 2009). Elegami et al. established the efficacy of consuming roasted seeds of *R. vesicarius* for curing dysentery (Elegami et al. 2001). In traditional medicine, the entire plant might be beneficial for treating hepatic diseases, tumors, constipation, poor digestion, dyspepsia, flatulence, bronchitis, asthma leucoderma, scabies, piles, and bacterial infections (El-Hawary et al., 2011, Rahman et al., 2004).

Whole plant extracts contain a variety phytochemicals such as catechins, naringenin, ferulic acid ester, luteolin glucosides, and apigenin (Hariprasad & Ramakrishnan, 2012). The species is also a source of proteins, lipids, vitamins (especially vitamin C), carotenoids, and organic acids (Alfawaz, 2006). In addition, roots are rich in anthraquinones with **proven** antibacterial, purgative, and laxative properties (Mostafa et al., 2011).

Plant polyphenols have gained attention for positive effects on human health and harmful effects on the digestive system. Phenolic compounds are typically poorly absorbed from the gastrointestinal (GI) tract and may inhibit digestive enzymes involved degradation of lipids, saccharides, and proteins involved in the degradation of lipids, saccharides, and proteins (Cirkovic Velickovic & Stanic‐Vucinic, 2018). However, the negative influences of phenolics and flavonoids on the hydrolysis of energy-rich foods (lipids and saccharides) might be beneficial for diabetics and in weight-control diets. Moreover, inhibiting pepsin activity will protect the gastric mucosa and thus help treat peptic ulcers and upper GI diseases (Pearson & Roberts, 2001). Still, inhibition of protein digestion of the proteins because of reduced utilization of essential amino acids (Cirkovic Velickovic & Stanic‐Vucinic, 2018).

Inhibiting α-amylase activity can be used to lower postprandial blood glucose levels in diabetic patients (Kumar et al., 2013). Conversely, angiotensin-converting enzyme (ACE) catalyzes the conversion of angiotensin I to II, a potent vasoconstrictor, and deactivates bradykinin, a vasodilator, and significantly influence blood pressure. Inhibiting ACE is now considered the therapeutic target in hypertension management.

Prebiotics are preparations that lead selectively to modification of the host microbiota, improve GI function, and promote metabolic, mental, and bone health. Dietary fibers, particularly oligosaccharides, such as galacto-oligosaccharides, fructo-oligosaccharides, and inulin, are well-defined prebiotics (Rezende et al., 2021). Polyphenols are proposed as candidate prebiotics based on their interaction with host microorganisms (Kumar Singh et al., 2019). Moreover, prebiotics may prevent or reduce hypertension (Pearson & Roberts, 2001). Probiotics and prebiotics, along with antidiabetic drugs, also enhance insulin sensitivity and glycemic control in mice (Stenman et al., 2015).

*E. coli* Nissle 1917, a gram-negative bacterium, shows probiotic characteristics. This bacterial strain is the most widely examined worldwide. *E. coli* Nissle 1917 has been applied as the bioactive pharmaceutical component in a licensed medicinal product for about 100 years. This products is in Germany and other countries. Activities of novel probiotics are often attributed to this bacterial strain (Scaldaferri et al., 2016).

However, no studies regarding the prebiotic potential of the ruby dock on different probiotic bacteria are reported to date to the best our knowledge. This study investigated the prebiotic activity of *R. vescarius* extracts and isolated compounds on *E. coli* Nissle 1917 in vitro. Second, the effect of their inhibitory activity on α-amylase, protease, and ACE enzymes was assessed.

## 2 Material and methods

### 2.1 General procedures

HR-ESI-MS was determined using LC-MS–IT–TOF (Shimadzu, Tokyo, Japan). Infrared spectra were recorded on Mattson 5000 FTIR (England) in the KBr pellet. The 1D and 2D spectral analyses were performed on the Bruker Ascend ^TM^ spectrometer (400 MHz) instrument. Using solvent peak as an internal standard, coupling constants (*J*) values were expressed in Hertz (Hz). Chromatographic separation was performed on a silica gel column (60–200 µm; Merck, Germany) and Sephadex LH-20 (Amersham Pharmacia Biotech). Precoated plates with silica gel 60 GF _254_ were used for TLC (Merck or Machery-Nagel, Germany) which was visualized by ultraviolet light and by using vanillin/sulfuric acid spraying reagent, then heating the plates at 110 °C for 5–10 min.

### 2.2 Plant material

*Rumex vesicarius* L*.* (Polygonaceae) was collected in April 2020 from the south area of the El-Tih desert, Sinai Peninsula, Egypt. Authentication of the plant was done by St. Cathrine Herbarium staff members. The fresh roots were separated from the whole plant and air-dried at room temperature in shade. A specimen with a voucher code Rv 06 Mansoura 3, was kept in the Department of Pharmacognosy in the Faculty of Pharmacy, Mansoura University.

### 2.3 Extraction and isolation

The dried root parts (600 g) were powdered and macerated with 90% MeOH at room temperature. The dried extract (60.2 g) was fractionated with petroleum ether, methylene chloride, ethyl acetate, and *n*-butanol (Fig. S28). The petroleum ether fraction (2.59 g) was chromatographed over a column of silica gel (2 × 50 cm) packed with petroleum ether. The column was run with a petroleum ether-ethyl acetate gradient from (100:0 to 93:7 v/v) to give 5 fractions (1–5). Fraction 1 was eluted with 100% petroleum ether and purified by crystallization to afford compound **1**. Fraction 2 was eluted with petroleum ether-ethyl acetate (99.5:0.5) and then subjected to a silica gel column (46 × 1.5 cm), using isocratic elution with petroleum ether-ethyl acetate (99:1) to afford compound **2**. Fraction 3 was eluted with petroleum ether-ethyl acetate (99:1) and then purified by crystallization to provide compound **3**. Fraction 4 was eluted with petroleum ether-ethyl acetate (94:6) and purified by crystallization to give compound **4**. Fraction 5 was eluted with petroleum ether-ethyl acetate (93:7) and subjected to crystallization to afford compound **5** in pure form.The ethyl acetate fraction (8.37 g) was chromatographed over a silica gel column (4 × 51 cm) prepared in methylene chloride. The column was run with methylene chloride-methanol (99:1 to 85:15) to give 4 fractions (1–4). Fraction 1 was purified by crystallization to give compound **6**. Fraction 2 was re-chromatographed on normal phase silica gel CC using isocratic mobile phase of petroleum ether-ethyl acetate (50:50), followed by further purification of the subfractions 25–27 on Sephadex LH 20 using methanol 100% as an eluent to isolate compound **7** in pure form. Fraction 3 was subjected to Sephadex LH 20 for further purification using methanol 100% as mobile phase to isolate compound **8**.

### 2.4 Determination of oxalic acid in extract and fractions

Oxalic acid concentrations in extracts prepared from the roots of ruby dock were determined adopting the indole reagent method. Briefly, 2 mL of each extract (1 mg/mL) was mixed with 2 mL indole reagent (1 mg/1mL). All test tubes were transferred into the water bath for 45 min at 90 °C, followed by cooling to room temperature, and then absorbance was recorded at 525 nm by spectrophotometer. Standard oxalic acid solution with different concentrations ranging from 0.1 to 1 mg/mL was prepared. The blank is this assay included the 2 mL of sulfuric acid (1 N) instead of the sample (Naik et al., 2014).

### 2.5 Assessment of biological activities

#### 2.5.1 Determination of total phenolic contents

The total phenolic content was determined by utilizing Folin-Ciocalteu reagent and Gallic acid standard. Briefly, 500 µL of Folin-Ciocalteu reagent (50%) was mixed in a test tube including 100 µL of each extract (1 mg/mL) and 3.5 mL deionized water, followed by incubation of mixture at room temperature for 120 min and then 500 µL of Na_2_CO_3_ (20%) was added. The mixture was then re-incubated for 60 min in a dark room. The absorbance of this mixture was measured at 720 nm by using a spectrophotometer. The total phenolic contents are expressed as gallic acid equivalents (mM GAE/g) (Singleton et al., 1999).

#### 2.5.2 Assessment of the prebiotic potential of isolated compounds

##### 2.5.2.1 Bacterial strains and growth conditions

*Escherichia coli* K12 (K_12_) was chosen as a representative enteric species, and *E. coli* Nissle 1917 (EcN) was chosen as a representative probiotic strain. Both strains were obtained from the bacterial strain collection in Food Microbiology Laboratory (Dairy department, Faculty of Agriculture, Mansoura University, Egypt). An overnight culture of both strains was activated in nutrient broth (NB) (BD Difco™, Bacton, Dickinson and Company Sparks, MD, USA) at 37 °C for 16–18 h. The culture was diluted on test days in buffered peptone water to a final concentration of 1.5 × 10^8^ colony forming units (CFU)/mL cell counts were assessed by pour plate method, one mL of a suitable dilution was incubated on nutrient agar at 37 °C for 24 h. colonies were then counted and converted to CFU/mL (Dawood et al., 2021).

##### 2.5.2.2 Growth kinetic curves of *Escherichia coli* Nissle 1917

An aliquot (100 µL) of EcN was inoculated into NB (BD Difco ^TM^) and incubated at 37 °C for 24 h. An overnight culture of EcN was further streaked onto nutrient agar and incubated for 48 h at 37 °C. The EcN culture was diluted in sterile saline solution (0.85%) on the day of assay to adjust the final cell count to 1.5 × 10^8^ CFU/mL. Bacterial growth was determined in NB supplemented with individual pure compounds (**1**–**8**) or extracts (methanol, petroleum ether, and ethyl acetate) at 2 mg/mL. Growth kinetics of EcN were monitored spectrophotometrically by OD_600_ every of 60 min at 37 °C (Pacheco-Ordaz et al., 2018). DMFit ver. 2.1 an Excel add-in was used to determine: doubling time (T_d_), lag time, maximum growth at the stationary phase (Y _max_, _OD_), and specific growth rate (*µ*_max_, _OD_).

##### 2.5.2.3 Prebiotic index of *Escherichia coli* Nissle 1917

Prebiotic index (PI) was estimated as previously described (Dawood et al., 2021). *E. coli* Nissle 1917 was used as the representative probiotic strain. Aliquots containing 10^7^ CFU of an overnight culture of EcN were inoculated into separate tubes containing NB. Two mg/mL of extracts or isolated phenolic compounds or 2 mg/mL of glucose as a positive control was added, and growth of EcN was assessed as the number of viable colonies forming units (CFU)/mL after incubation for 24 h using the pour method on nutrient agar, followed by incubation at 37 °C for 24 h. PI is the ratio of growth of EcN in NB supplemented with individual compounds or extracts to growth on glucose. A PI greater than one indicates a positive influence on the growth of EcN. PI was calculated as follows:

(1) $$\mathrm{PI}=\frac{\mathrm{CFU}\;of\;EcN\;in\;Phenolic\;Compounds\;(as\;prebiotic)}{\mathrm{CFU}\;of\;ECN\;in\;glucose\;(as\;control\;carbohydrate)\;}$$

##### 2.5.2.4 Prebiotic activity score

Prebiotic activity scores (P_score_) were calculated as previously described (Dawood et al., 2021):

(2) $$\mathrm{Pscore}=\frac{\left(Log\;P24-\;Log\;P0\right)\;PC}{\left(Log\;P24-\;Log\;P0\right)\;Glucose\;}-\frac{\left(Log\;E24-\;Log\;E0\right)\;PC}{\left(Log\;E24-\;Log\;E0\right)\;Glucose\;}$$

where P_score_ is prebiotic activity score; Log P is log of CFU/mL after 24 h (P_24_) and zero time (P_0_) of incubation at 37 °C. Log E is log of CFU/mL of K_12_ at zero time (E_0_) and 24 h (E_24_) of culture on glucose and pure compounds. Individual pure compounds or extracts that induce a high value of P_score_ improve EcN growth compared with the growth rate of EcN on glucose. Support of K12 growth by pure compounds or extracts theoretically should be low compared to growth of K_12_ on glucose.

#### 2.5.3 Determination of the inhibitory activity of enzymes

##### 2.5.3.1 Determination of the effect of extracts or isolated compounds on inhibition of α-amylase

α-amylase inhibition was determined as previously reported (Hu et al., 2013). Briefly, 200 µL of 20 mM sodium phosphate buffer (pH 7.0 with 6 mM NaCl), including α-amylase (10 U/mL) and 200 µL of sample (2 mg/mL), was incubated at 37 °C for 45 min. A potato starch solution (400 µL; 0.5%) was added to each sample and incubated at 37 °C in a shaking water bath (100 rpm) for 10 min. The enzymatic reaction was stopped with dinitrosalicylic acid color reagent. Screw cape tubes were transferred to a water bath at 100 °C for 10 min, then cooled to room temperature. Finally, samples were diluted with 3 mL of deionized water (3 mL). Absorbance of diluted reaction mixtures was measured spectrophotometrically at 540 nm (Spectro UV–VIS Auto, UV2602, Labomed, USA). Test and control samples were compared and expressed as percentage inhibition of α-amylase. Acarbose (5 µg/mL) was used as a positive control, and samples in buffer without test compounds or extracts were included as negative controls.

##### 2.5.3.2 Determination of the effect of extracts or isolated compounds on inhibition of protease

Protease used the protocol of Hu et al. (2013). Briefly, 100 µL of pepsin solution (1 mg pepsin/1 mL of 0.02 N HCl) was mixed with 0.5 mL of 10 mg/mL bovine serum albumin and 0.5 mL of extracts or pure compounds (2 mg/mL) and incubated for 30 min at 37 °C. TCA (0.9 mL 5%) was added to stop the reaction, followed by centrifugation (4800*g*, 15 min). Protein concentration in the final supernatant was determined using Coomassie (Bradford) protein assay kits. Pepstatin A (0. 5 µg/mL) was used as a positive control.

##### 2.5.3.3 Determination of the effect of extracts or isolated compounds on angiotensin-converting enzyme inhibitory activity

Lyophilized aqueous extract (whey) obtained from yogurt enriched with extracts or individual compounds at 2 mg/mL was used. The inhibitory activity was estimated spectrophotometrically as reported by Cavalheiro et al. (2020) with slight modification. The method depends on hipuryl-histdylleucine (HHL) cleavage to hippuric acid by ACE. HHL (3.8 mM) was diluted in 0.1 M borate buffer (200 μL; pH 8.3), including 0.3 M NaCl, then 35 μL of sample solution (2 mg/mL) was added, and the mixture incubated for 10 min at 37 °C. The reaction was initiated by adding 20 μL of ACE solution (0.1 U/mL in borate buffer) and allowed to proceed for 30 min at 37 °C. HCl (250 μL of a 1 M solution) was then added to stop the reaction. Hippuric acid was extracted by vigorous mixing with 1.5 mL of ethyl acetate (1.5 mL) for 30 s and centrifugation at 1200*g* for 10 min. Subsequently, 1 mL of supernatant (ethyl acetate) was collected in a new tube and placed in boiling water for 30 min to eliminate the solvent. The remaining hippuric acid residue was dissolved in 1 mL of distilled water, and absorbance was measured at 228 nm using distilled water as a blank. All samples were analyzed in triplicate. Captopril (0.008 mg/mL) was used as a positive control, and lyophilized aqueous extract (whey) obtained from plain yogurt was used as a negative control. The calculation of ACE inhibitory activity was determined as follows:

(3) $$\text{ACE inhibition}\;\left(\%\right)=\left[1\;-\;\left(A-C\right)/\left(B-D\right)\right]\times 100$$

where A is absorbance with the sample, HHL, and ACE; B is absorbance with HHL and ACE without sample; C is absorbance with sample and HHL; D is absorbance with HHL without sample and ACE. IC_50_ values (concentrations of extracts or compounds that caused a 50% reduction in ACE activity) were estimated by regression of OD_228_ obtained with different concentrations of extracts, pure compounds, and positive control.

### 2.6 Statistical analysis

Mean percentages of growth of EcN, PI, P_Score_, and protease, α-amylase, and ACE inhibition from 3 independent repeats were analyzed using one-way analysis of variance via SAS 2000. Pairwise comparisons between means were estimated by Duncan’s Multiple Range Test when main effects were significant. Principal component analysis (PCA) used unsupervised clustering to recognize outliers and trends in data sets. All parameters were regressed onto a plot using partial least squares regression (PLSR).

## 3 Results and discussion

### 3.1 Isolation and identification of compounds

**Fig. 1:**
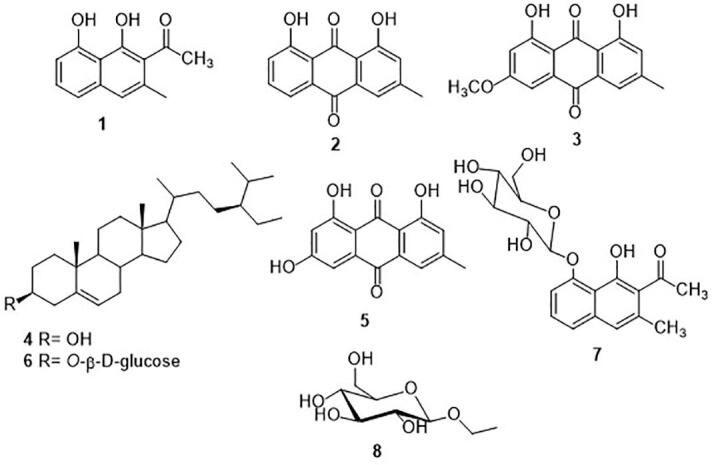
Structures of the isolated compounds from *Rumex vesicarius* L*.*

Eight known compounds were separated from the petroleum ether and the ethyl acetate extracts of *Rumex vesicarius* (Fig. 1). Nepodin (**1;** 50 mg) was characterized by analyzing the spectroscopic data and comparing them with literature (Colegate et al., 1985). The APT and ^1^H NMR spectral data of **2**, **3,** and **5** are in full agreement with reported data for chrysophanol (5 mg), physcion (6 mg), and emodin (10 mg), respectively (Danielsen et al., 1992). Physical and chemical properties, IR and ^1^H NMR spectral data of **4** (3 mg) were identical to those reported for *β*-sitosterol (Chaturvedula and Prakash, 2012, Rajput and Rajput, 2012). The chemical and physical properties, ^13^C NMR and IR absorbances of **6** were consistent with those published for *β*- sitosterol 3-*O*-*β*-d-glucoside (Silverstein & Bassler, 1962). Compound **7** was identified as 6-methyl-7-acetyl-1, 8-dihydroxy naphthalene-1-*O*-*β*-d-glucoside (40 mg) by comparison of its spectral data with values found in the literature (Zhang et al., 2012). Compound **8** was identified as ethyl *β*-d-glucopyranoside (5 mg) by comparison of its ^1^H NMR and APT data with those of Li et al. (2003). Compound **8** hasn’t been isolated previously from the ruby dock.

### 3.2 Determination of oxalic acid in total extract and fractions

The oxalic acid was present at 4.37, 3.22, and 2.57 mg/100 g in total extract, ethyl acetate fraction, and petroleum ether, respectively. Concentrations in extracts or fractions are considered low compared with other vegetables (Alfawaz, 2006) and are consistent with a previous report that the lowest of oxalic acid content is commonly found in roots while the highest levels are encountered in leaves (Naik et al., 2014). In our study, levels of oxalic acid are lower than reported for sorrel leaf (Alfawaz, 2006). Oxalic acid is an important factor that decreases the bioavailability of several components of human diets.

### 3.3 Total phenolic contents

Total phenolic contents of total extract and fractions (petroleum ether and ethyl acetate) prepared from roots of the ruby dock were measured by Folin-Ciocaltea assay (Table S1). Total phenolic content ranged from 59.82 to 80.24 mg GAE/g extract in the root extract/fractions. Extraction solvent had a notable (*P* < 0.05) influence on total phenolic content; the ethyl acetate fraction contained the higher concentrations. Our finding is consistent with the previous report (Elzaawely & Tawata, 2012), who reported that an ethyl acetate fraction from of *R. dentatus* root exhibited higher concentrations than other fractions. Total phenolic root extracts/fractions content was also higher than content in leaf extracts/fractions.

### 3.4 Assessment of the prebiotic potential of extracts and pure compounds

#### 3.4.1 The influence of individual pure compounds on growth kinetics of *Escherichia coli* Nissle 1917

EcN grew at different rates (µ_max_) in controls and media supplemented with pure compounds (Table 1). Still, the µ_max_ and Y_max_ of EcN in supplemented media were significantly (*P <* 0.05) higher than the control. All tested compounds promoted the growth of EcN compared with growth in controls. Compounds **2**, **3**, **4**, **6**, **7**, and **8** also significantly (*P <* 0.05) decreased lag phase l (Table 1). Doubling times varied from 39.71 min for compound **4** to 74.68 min in control (Table 1). The lowest value of T_d_ was observed in **4** supplemented media.

**Table 1:** Effects of extracts and pure compounds on growth kinetics of EcN.

Treatments	Lag (min)	µ _max (OD /_ min)	Y_max (OD)_	T_d_ (min)
Control	240 ^a^	0.009 ^e^ ± 0.001	1.5 ^g^ ± 0.015	74.68 ^a^ ± 4.02
RT	60 ^c^	0.014 ^bc^ ± 0.001	2.17 ^b^ ± 0.02	50.6 ^cde^ ± 3.13
RP	60 ^c^	0.017 ^a^ ± 0.002	2.4 ^a^ ± 0.02	40.88 ^e^ ± 1.21
RE	60 ^c^	0.014 ^b^ ± 0.002	2.25 ^b^ ± 0.03	49.56 ^ed^ ± 1.88
**1**	240 ^a^	0.01 ^de^ ± 0.001	1.62 ^f^ ± 0.04	67.23^ab^ ± 3.97
**2**	180 ^b^	0.012 ^bcd^ ± 0.001	1.82 ^cd^ ± 0.021	56.42^bcd^ ± 2.38
**3**	180 ^b^	0.012 ^bcde^ ± 0.001	1.74 ^de^ ± 0.04	59.39 ^bcd^ ± 3.75
**4**	60 ^c^	0.017 ^a^ ± 0.001	2.43 ^a^ ± 0.02	39.71^e^ ± 1.23
**5**	240 ^a^	0.011^cde^ ± 0.001	1.69 ^ef^ ± 0.024	62.53 ^bc^ ± 4.95
**6**	180 ^b^	0.014 ^bc^ ± 0.001	1.88 ^c^ ± 0.01	51.37^cde^ ± 4.5
**7**	180 ^b^	0.011 ^cde^ ± 0.001	1.71 ^e^ ± 0.035	62.34 ^bc^ ± 2.85
**8**	180 ^b^	0.012 ^bcde^ ± 0.001	1.80 ^cd^ ± 0.015	57.98 ^bcd^ ± 4.36

Note: (*n* = 3; average ± Standard diffusion) values in rows with different superscripts (a-d) have significant differences at *P<*0.05. RT (total extract), RP (petroleum ether fraction), RE (ethyl acetate fraction), **1** (nepodin), **2** (chrysophanol), **3** (physcion), **4** (β-sitosterol), **5** (emodin), **6** (β-sitosterol 3-*O*-*β*-d-glucoside), **7** (6-methyl-7-acetyl-1,8-dihydroxy naphthalene-1-*O*-*β*-d-glucoside) and **8** (ethyl *β*-d-glucopyranoside).

Several probiotic bacterial strains produce enzymes (e.g., β-galactosidase, *α*-rhamnosidase, and β-glucuronidase) that utilize pure compounds as carbon sources and effectively promote the use of alternative nutrients (García-Ruiz, Bartolomé, Martínez-Rodríguez, Pueyo, Martín-Álvarez, & Moreno-Arribas, 2008). In particular, species in the genus *Lactobacillus* have a gallate decarboxylase enzyme and convert gallic acid to pyrogallol (Jimenez et al., 2013). This metabolite is then hydrolyzed to *cis*-aconitate and enters the Krebs cycle. Gallic acids can also be metabolized to oxaloacetate and pyruvate (Bhat et al., 1998). Many enzymes, such as hydrogenases, decarboxylase, dehydrogenases, esterases, dehydroxylase, and isomerases, degrade pure compound structures into C3-carbon intermediates (Selma et al., 2014).

#### 3.4.2 Effects of extracts on *Escherichia coli* Nissle 1917

**Fig. 2:**
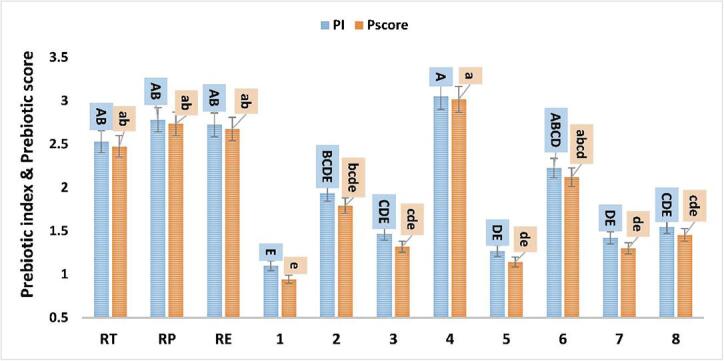
Prebiotic index and prebiotic activity score of EcN paired with different extract and individual phenolic compounds. RT (total extract), RP (petroleum ether fraction), RE (ethyl acetate fraction), **1** (nepodin), **2** (chrysophanol), **3** (physcion), **4** (*β*-sitosterol), **5** (emodin), **6** (*β*-sitosterol 3-*O*-*β*-d-glucoside), **7** (6-methyl-7-acetyl-1,8-dihydroxy naphthalene-1-*O*-*β*-d-glucoside) and **8** (ethyl *β*-d-glucopyranoside). Different letters (upper cases) were significant (*P <* 0.05) *vs.* prebiotic index and different letters (lower cases) were significant (*P <* 0.05) *vs.* prebiotic activity score.

Extracts (Rum total, Rum Pet, and Rum EA) caused a higher growth compared with the pure compounds, except for compound **4** (Fig. 2). Rum Pet accelerated the growth of EcN compared with compounds **1**, **2**, **3**, and **5**. Further, combining individual pure compounds in Rum EA enhanced growth (Table 1). Pacheco‐Ordaz et al. reported contrary results where pure combined compounds, decreased growth rates of *Lactobacillus rhamnosus* and *L. acidophilus* compared with individual compounds (Pacheco-Ordaz et al., 2018). These studies used protocatechuic acid in combination with either catechin or gallic acid*.* Previous studies of pure compound combinations with antimicrobial activity against pathogenic microorganisms or stimulative probiotic action are scarce. Extracts of green tea enhanced survival of *Bifidobacterium ainimalis* B94 compared to saline solution (de Lacey et al., 2014). Conversely, Tabasco et al. reported that grape seed extract enriched with gallic acid (5.5 mg mL^−1^) and catechin (25 mg mL^−1^) adversely affected the growth of various *Lactobacillus* strains.

#### 3.4.3 Prebiotic index of pure compounds and extracts

The highest PI was recorded for EcN paired with compound **4**, Rum Pet, Rum EA, and Rum Total (3.05 ± 0.47, 2.78 ± 0.42, 2.72 ± 0.47, and 2.53 ± 0.40, respectively). In contrast, low PI (*P <* 0.05) were observed with compounds **1** and **5** (0.94 ± 0.24 and 1.14 ± 0.20, respectively) (Fig. 2). Negative or low PI values are obtained if a probiotic strain grew less well in the presence of a prebiotic (extracts or purified compounds) compared with glucose.

#### 3.4.4 Prebiotic activity score of pure compounds and extracts

EcN strain was chosen as probiotic in the present study, while *E.coli* K12 was used as enteric bacteria of P_Score_**_._** The P_score_ values showed in Fig. 2 were derived from the growth of EcN according to Eq. (2). All P_score_ values were positive indicating that the growth rate of EcN in different extracts or individual compounds were higher than *E.coli* K12 paired with the tested compounds. In this study (**4**, Rum Pet, Rum EA, and Rum total) were found to have a significant (*P <* 0.05) effect on the growth of EcN, comparable glucose. EcN paired with **4**, Rum Pet, Rum EA, and Rum total presented outstanding P_score_ values of about 3.02 ± 0.48, 2.73 ± 0.43, 2.68 ± 0.46, and 2.47 ± 0.40, respectively (Fig. 2).

PI and P_score_ values obtained with Rum Total, Rum Pet and Rum EA were significantly (*P <* 0.05) higher than for individual compounds (**1**, **3**, **5,** and **7**). Thus, a synergistic effect among isolated compounds toward EcN is likely. However, no statistically significant differences between PI and P_score_ of extracts and other individual compounds were found (Fig. 2).

No previous findings of the stimulating effects of purified phenolic compounds or extracts on *E. coli* Nissle 1917 have been reported, to the best of our knowledge. Previous studies were concerned with combining both polyphenolic compounds and *Lactobacillus* or *Bifidobacterium* (Piekarska-Radzik & Klewicka, 2021). Several phenolic compounds and their metabolites stimulate the growth of probiotics bacteria in the human gut (Piekarska-Radzik & Klewicka, 2021). Dolara et al. (2005) fed rats red wine powder that induced a significant increase in viability of *Lactobacillus* genus bacteria in rat feces. Similarly, Tabasco et al. (2011) showed that polyphenols, including flavan-3-ol, epicatechin, and catechin, in concentrations of 0.25–1.0 mg/mL, are growth activators for lactic acid bacterial strains. Further, when phenolic compounds were applied as individual compounds, they occasionally inhibit of growth of strains, such as *Lactobacillus casei* and *L. plantarum*. Coman et al. (2018) reported that ethanolic extracts of red fruit (different parts of elderberry, plum skin, and Italian red grape skin) stimulated the growth of *Lactobacillus paracasei* IMC 502 and *L. rhamnosus* IMC 501 alone or in combination. Additionally, stimulatory effects of phenolic compounds on probiotic strains may be paired with oxygen-scavenging abilities. Effective antioxidants produced by microbial activity may modify oxidative stress and alter GI microbiota composition by activating probiotic and inhibiting pathogenic bacteria (China et al., 2012).

#### 3.4.5 The inhibitory activity of extracts or isolated compounds against protease

**Fig. 3:**
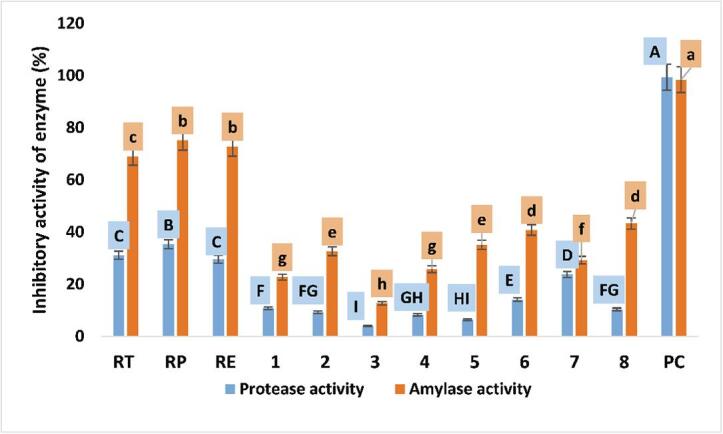
Effect of the PC combination (extracts) and individual PC on inhibitory activities of protease and α- amylase. RT (total extract), RP (petroleum ether fraction), RE (ethyl acetate fraction), **1** (nepodin), **2** (chrysophanol), **3** (physcion), **4** (*β*-sitosterol), **5** (emodin), **6** (*β*-sitosterol 3-*O*-*β*-d-glucoside), **7** (6-methyl-7-acetyl-1,8-dihydroxy naphthalene-1-*O*-*β*-d-glucoside), **8** (ethyl *β*-d-glucopyranoside) and PC (positive control). Different letters (upper cases) were significant (*P <* 0.05) *vs.* inhibitory activity of protease and different letters (lower cases) were significant (*P <* 0.05) *vs.* inhibitory activity of α- amylase.

All extracts and individual compounds at 2 mg/mL significantly (*P <* 0.05) reduced pepsin activity compared with control (Fig. 3). The rate of decrease in pepsin activity in the presence of extracts was significantly (*P <* 0.05) higher than for individual compounds (Fig. 3). Pepstatin, the positive control, showed inhibitory activity greater than for any extracts or isolated compounds. Similarly, a decline in protein digestibility was associated with phenolic compounds (He et al., 2007, Karaś et al., 2017, Renard et al., 2017).

Consistently, He et al. (2007) reported that tea polyphenols inhibited trypsin and pepsin. Tea is a rich source of epigallocatechin, epicatechin-3-gallate, gallocatechin, epicatechin, epigallocatechin, and catechins (Samuels et al., 2013). Also, pepsin inactivated by anthocyanin and curcumin in the black raspberry extract.

Reduced digestion of dietary protein induced by phenolic compounds may be partly associated with destabilizing enzyme conformation and orientation of substrate-binding/proper position/catalytic residues, resulting in enzyme inactivation (Cirkovic Velickovic & Stanic‐Vucinic, 2018). Also, phenolic compounds might block catalytic sites, substrate-binding sites, or both, thus decreasing proteolytic activity (Cirkovic Velickovic & Stanic‐Vucinic, 2018). Further, pure compounds may act as allosteric regulators of pepsin activity (Cirkovic Velickovic & Stanic‐Vucinic, 2018).

#### 3.4.6 The inhibitory activity of extracts and isolated compounds against α- amylase

Extracts and compounds at of 2 mg/mL caused (*P <* 0.05) a decrease in the activity of α-amylase activity. The rate of α-amylase activity reduction for compounds was significantly (*P <* 0.05) lower than for extracts (Fig. 3). Further, acarbose inhibition was greater than for either extracts α- amylase by extracts or individual compounds. The rate of α- amylase activity reduction was higher than for pepsin activity (Fig. 2), as consistent with the previous report (He et al., 2007), who found that tea polyphenols resulted in higher inhibition of amylase (61%) than pepsin, trypsin, or lipase (32%, 38%, and 54%, respectively), due to its greater molecular weight. The inhibitory effects of extracts or individual compounds on digestion of energy-rich food (lipids and saccharides) may be considered as beneficial, primarily diet for diabetes or weight-control diets (Cirkovic Velickovic & Stanic‐Vucinic, 2018).

Diabetes is a common disease that sickens people in this century. The number of diabetic patients is increasing and demanding more attention in the public and medical sphere (American Diabetes, 2018). Type 2 diabetes mellitus (T2D) is the predominant form, and is anticipated to reach pandemic levels. This disease in Egypt is associated with various factors, including sedentary lifestyle, stress, unhealthy food, and aging. Insulin resistance is the main cause of hyperglycemia. Targeting this process can reduce glucose levels related to diabetes and diabetic complications (Chandrasekhar et al., 2021, Ha et al., 2014, Lee et al., 2012, Melucci et al., 2018, Xie et al., 2019). Previously, an extract of *R. obtusifoliu*s displayed elevated levels of phenolic compounds, tannins, and flavonoids. These compounds could underlie antidiabetic effects of *R. obtusifoliu*s. Flavonoids could control the activity of rate-limiting enzymes in carbohydrate metabolism pathways, and might act as insulin secretagogues that enhance glucose uptake in peripheral tissues (Aghajanyan et al., 2018). Reddy et al. also indicated that concentrations of an ethanolic extract of ruby dock significantly reduced blood glucose level in streptozotocin-induced rat, due to its systemic influences, including pancreatic function.

#### 3.4.7 Inhibition of angiotensin-converting enzyme

**Fig. 4:**
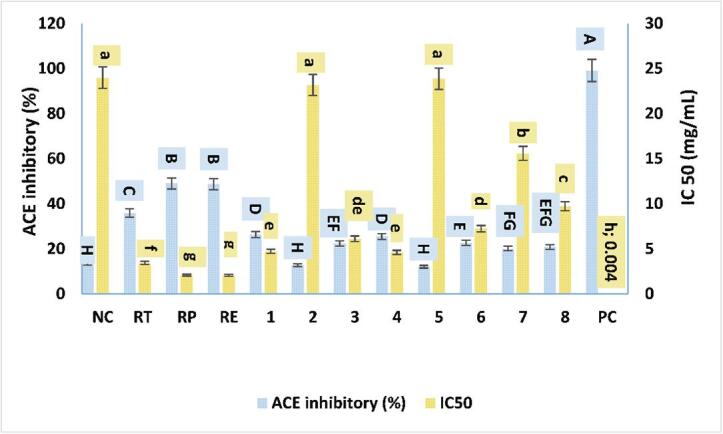
Effect of the PC combination (extracts) and individual PC on inhibitory activities of ACE (%) and IC_50_ . NC (negative control), RT (total extract), RP (petroleum ether fraction), RE (ethyl acetate fraction), **1** (nepodin), **2** (chrysophanol), **3** (physcion), **4** (*β*-sitosterol), **5** (emodin), **6** (*β*-sitosterol 3-*O*-*β*-d-glucoside), **7** (6-methyl-7-acetyl-1,8-dihydroxy naphthalene-1-*O*-*β*-d-glucoside), **8** (ethyl *β*-d-glucopyranoside) and PC (positive control). Different letters (upper cases) were significant (*P <* 0.05) *vs.* inhibitory activity of ACE (%) and different letters (lower cases) were significant (*P <* 0.05) *vs.* inhibitory activity of ACE (IC_50_).

Angiotensin -1- converting enzyme- inhibitory peptides are bioactive peptides with antihypertensive properties. Those peptides are generated as metabolites of bacterial peptidases and proteinases that have been widely detected in several dairy products (Gandhi & Shah, 2014). The modification in percentage inhibition of ACE activities and corresponding values of IC_50_ for yogurt enriched with compounds or extracts after 7 days of refrigerated storage are shown in Fig. 4. The addition of extracts or individual compounds at 2 mg/mL led to a significant (*P <* 0.05) increase in ACE inhibitory activity compared with control. The ACE-inhibitory activities of yogurt supplemented with compound **1** have the highest compared with other isolated compounds, while yogurt supplemented with **5** presented the lowest inhibitory activity of ACE compared with other compounds (Fig. 3). ACE inhibitory activity of extracts was significantly (*P <* 0.05) higher than all tested individual compounds (Fig. 4). The IC_50_ were estimated among these supernatants obtained from yogurt enriched with pure compounds varied from 2.07 mg/mL for Rum EA to 23.86 mg/mL associated with compound **5** (Fig. 3). Consistently, Al Shukor et al. (2013), reported that 22 phenolic compounds increased inhibition of ACE-activity. Docking studies suggested inhibition of ACE by binding with zinc ion that stabilized the active site for other interactions. Phenolic compounds, such as pyrogallol and resveratrol, can reduce ACE by binding with amino acid residues at the active site, subsequently blocking enzyme catalysis. All treatments (extracts or isolated compounds) presented significantly (*P* < 0.05) higher values of IC_50_ than captopril (4 µg/mL), the positive control. Therefore, yogurt supplementation of extracts or isolated compounds, except compounds **2** and **5**, would be sufficient to induce significant inhibition of ACE. Binding between milk proteins and phenolic compounds could reduce the bioavailability of phenolic compound in yogurt as ACE inhibitors (Baba et al., 2014).

Phenolic compounds isolated from various plants are efficient ACE inhibitors in vitro (Dong et al., 2011). However, these substances exhibit low solubility and may have limited bioavailability. Nevertheless, experiments with spontaneously hypertensive rats indicate that phenolic compounds, such as ferulic acid and quercetin, can lower blood pressure *in vivo* (Alam et al., 2013, Häckl et al., 2002).

An extract of *Rumex acetosa* L. (ERA) enhanced phosphorylation of oxide synthase (eNOS) and protein kinase B (Akt). This activity was inhibited by LY294002 and wortmannin, indicating the involvement of the PI3-kinase/protein kinase B (Akt) pathway in oxide synthase phosphorylation. Moreover, intravenous administration of ERA to anesthetized rats reduced arterial blood pressure in a dose-dependent manner through stimulation of nitric oxide-nitric oxide synthase. ERA stimulates vasorelaxation *via* signal transduction in endothelium-dependent vasodilatation. The process involves two-stage signaling. First, endothelial cells are activated by Ca^2+^-eNOS-NO and P13-kinase/protein kinase B signaling. Second, muscular NO-sGC-cGMP signaling is activated (Sun, Su, Jin, Zhou, Sun, Wen, et al., 2015). Further, ERA exhibits endothelium-independent modulation of calcium entry through voltage-gated Ca^+2^ channels and release from internal calcium stores. These actions likely underlie its antihypertensive influence in normotensive and salt-induced hypertensive rats (Qamar et al., 2018). Ahmad et al. (2016) reported that an essential oil isolated from *R. hastatus* inhibits cholinesterase activity. Anticholinesterase-like substances are known to reduce vascular resistance and blood pressure.

#### 3.4.8 Principal component analysis of biological activities of isolated compounds

**Fig. 5:**
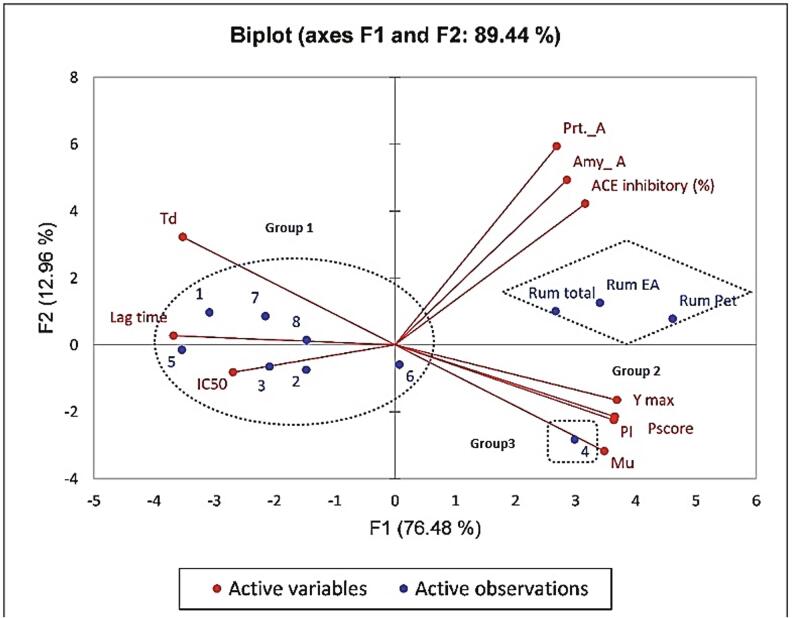
Principal component (PC) analysis biplot for the influence of phenolic compounds on prebiotic parameters (µ_u_, Y_max_, lag time, and T_d_) and inhibition of enzymes (α-amylase, protease, and ACE).

PCA of prebiotic potential of isolated compounds and related enzymes activity explained 89.44% of the variability with 2 PC (Fig. 5). PC1 (76.48%) comprised µ_u_, Y_max_, PI, P_score_, inhibition of protease (Prt_A), inhibition of amylase (Amy_A), and ACE inhibition. These attributes ran counter to IC_50_ values for ACE inhibitory. PC2 (12.96%) included T_d_ and lag time (Fig. 5). Two-dimensional component plots were generated only for PC1 and PC2 due to maximum weighting for PC1. Three groups were identified. Group 1 was positioned on the left side of PC1, and groups 2 and 3 were positioned on the positive side of PC1. Group 1 is characterized by higher values of T_d_, lag time, and IC_50_, group 2 with higher values for inhibition of α-amylase, protease, and ACE, and group 3 with higher values for µ_u_, Y_max_, PI, and P_score_. PCA was a useful tool to differentiate among samples based on impacts to tested parameters.

Inhibition of α-amylase, proteases, and ACE by individual phenolic compounds are often described, yet little information about their interactions and potential antagonistic or synergistic effects is available. Phenolic content of the ethyl acetate fraction, includes β-sitosterol 3-*O*-*β*-d-glucoside (**6**), 6-methyl-7-acetyl-1,8-dihydroxy naphthalene-1-*O*-*β*-d-glucoside (**7**), and ethyl *β*-d-glucopyranoside (**8**); phenolic contents of the petroleum ether fraction, includes nepodin (**1**), chrysophanol (**2**), physcion (**3**), β-sitosterol (**4**), emodin (**5**). These compounds showed synergistic effects, where ethyl acetate and petroleum ether fractions exhibited the greatest inhibition of α-amylase, proteases and ACE compared with total extract or isolated compounds.

No findings are available, to the best of our knowledge, regarding synergistic or antagonistic effects of phenolic compounds on enzymes inhibition. However, studies report synergistic/antagonistic effects of plant constituents on antioxidant activities. Hajimehdipoor et al. (2014) showed the combination of rutin, gallic acid, and quercetin in mixtures 1 and 2, including gallic acid, caffeic acid, and quercetin, display significant synergism (55.2% and 59.4%, respectively). However, addition of quercetin to mixture 1 reduced this effect compared with the combination of caffeic acid and gallic acid that showed an increased effect (137.8%). Hence, binary combinations of the above compounds are preferred.

#### 3.4.9 Comparison of prebiotic properties and enzyme inhibition

**Fig. 6:**
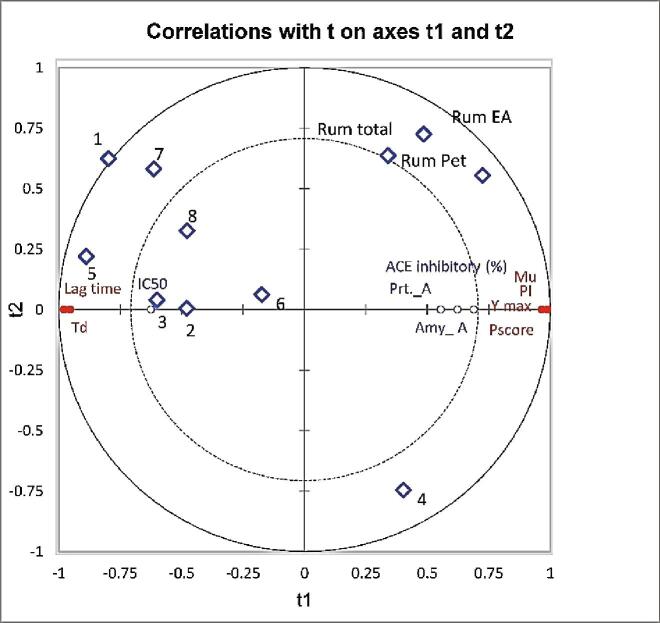
Partial least Square Regression (PLSR) biplot correlating prebiotic potential properties (red dots) and the rate of enzymes inhibition (blue dots) of 11 samples (rhombus)

Partial Least Squares Regression (PLSR) is particularly useful when many explanatory variables, probably correlated, are assessed. PLSR was used to examine relationships among prebiotic properties (PI, P_score_, µ_u,_ Y_max_, Lag time, and T_d_) and inhibition of enzymes (α-amylase, protease, and ACE). Inhibition was positively correlated with PI, P_score_, µ_u_, and Y_max_ (Fig. 6), and negatively correlated with T_d_ and lag time. Further, T_d_ and lag time positively correlated with IC_50_ values (Fig. 6).

## 4 Conclusions

*R. vesicarius* is a remarkable source of bioactive compounds that might serve as candidates for treating diabetes mellitus and hypertension. Extracts and isolated constituents decreased the activity of α-amylase and increased ACE inhibition. Inhibition of α-amylase may be also beneficial for weight-control diets. Additionally, all extracts and individual compounds reduced pepsin activity. Thus, isolated compounds and extracts could protect against peptic ulcers. This study is the first to show such inhibitory effects for constituents of ruby dock. Impacts on enzymes were more pronounced for extracts than individual compounds. Synergistic associations among phytoconstituents in extracts may underlie this finding. In addition, extracts and individual compounds supported the growth of *E. coli* Nissle 1917. This report is also the first to verify ruby dock as a promising source of natural prebiotics.

## Declaration of Competing Interest

The authors declare that they have no known competing financial interests or personal relationships that could have appeared to influence the work reported in this paper.
