# Supplementary material for: Isolation and biological activities of compounds from Rumex vesicarius L. and their use as a component of a synbiotic preparation
Source: Food Chem X. 2022 Apr 9;14:100306. doi: 10.1016/j.fochx.2022.100306 (PMC9043391; doi:10.1016/j.fochx.2022.100306)
Supplement: Supplementary data 1 [file mmc1.docx]

**Isolation and biological activities of compounds from *Rumex vesicarius* L. and their use as a component of a synbiotic preparation**

Ahmed Elbermawi^a^, Mohamed Samir Darwish^b*^, Asmaa A. El-Awady^b^, Ahmed A. Zaki^a,c*^, Longxin Qiu ^d,e*^, Reham M. Samra^a^

*^a^Department of Pharmacognosy Faculty of Pharmacy, Mansoura University, Mansoura, 35516 Egypt.*

*^b^Dairy Department, Faculty of Agriculture, Mansoura University, Mansoura 35516, Egypt*

*^c^Department of Pharmacognosy, Faculty of Pharmacy, Horus University-Egypt, New Damietta 34518, Egypt.*

^d^ *Key Laboratory of Preventive Veterinary Medicine and Biotechnology, Longyan University, Longyan, 364012, P.R. China. ^e^ Fujian Provincial Key Laboratory for the Prevention and Control of Animal Infectious Diseases and Biotechnology, Longyan, 364012, P.R. China*

**Abstract**

The study evaluated prebiotic potential and the enzyme inhibition of extracts and isolated compounds of *Rumex vesicarius* (ruby dock), family Polygonaceae. Eight known compounds were identified in the roots of *R. vesicarius*. Extracts and compounds (**1** - **8**) increased the growth rate of *Escherichia coli* Nissle 1917 differentially compared to controls. The highest prebiotic index (PI) and activity score was recorded for EcN in the presence of compound **4,** followed by, in descending order, petroleum ether, ethyl acetate, and total methanol extracts. The compounds and extracts reduced protease, α-amylase, and angiotensin-converting enzyme activities. This inhibitory activity was positively correlated with PI, P_score_, µ_u_, and Y_max_. These findings suggest that *R. vesicarius* is a good source of potential prebiotic and can boost beneficial bacteria. It may also be considered promising for treatment of diabetes mellitus, controlling weight, and regulating blood pressure.

**Keywords:** Ruby dock, Prebiotic, α- Amylase, Angiotensin-converting enzyme, *E. coli* Nissle 1917, Protease

**Corresponding authors: Dr. Mohamed S. Darwish (email: msamir@mans.edu.eg); Dr. Ahmed A. Zaki (email:** [**ahmed.awad@fulbrightmail.org**](mailto:ahmed.awad@fulbrightmail.org)**); Dr. Longxin Qiu (email:** [**qlongxin@tom.com**](mailto:qlongxin@tom.com)**)**

List of contents

Figure S1. ^1^H-NMR spectrum (400 MHz, CDCl_3_) of Nepodin (**1)**

[Figure S2. ^13^C-NMR spectrum of](#_Toc476257013) Nepodin (**1)**

Figure S3. HRESIMS^-^ spectrum of Nepodin (**1)**

Figure S4. HSQC spectrum of Nepodin (**1)**

Figure S5. HMBC spectrum of Nepodin (**1)**

[Figure S6. ^1^H-NMR spectrum (400 MHz, CDCl_3_) of chrysophanol **(2) -**](#_Toc476257017)

[Figure S7. ^13^C-NMR spectrum of chrysophanol **(2)**](#_Toc476257018)

[Figure S8. ^1^H-NMR (400 MHz, CDCl_3_) spectrum of physcion **(3)**](#_Toc476257005)   [Figure S9. ^13^C-NMR spectrum of physcion **(3)**](#_Toc476257006)

Figure S10. HSQC spectrum of physcion **(3)**

Figure S11. IR spectrum of *β-*sitosterol **(4)**

[Figure S12. ^1^H-NMR spectrum (400 MHz, CDCl_3_) of *β*-sitosterol (**4(**](#_Toc476257024)

[Figure S13. ^13^C-NMR spectrum of *β*-sitosterol (**4(**](#_Toc476257024)

[Figure S14. HSQC spectrum of *β*-sitosterol (**4(**](#_Toc476257024)

[Figure S15. HMBC spectrum of *β*-sitosterol (**4(**](#_Toc476257024)

[Figure S16. COSY spectrum of *β*-sitosterol (**4(**](#_Toc476257024)

Figure S17. ^1^H-NMR spectrum (400 MHz, MeOD) of emodin (**5**)

[Figure S18. APT spectrum of](#_Toc476257018) emodin **(5)**

Figure S19. HRESIMS^-^ spectrum of emodin **(5)**

Figure S20. IR spectrum of *β*- sitosterol 3-*O*-*β*-D-glucoside **(6)**

Figure S21. ^13^C-NMR spectrum (400 MHz, d6-DMSO) of *β*- sitosterol 3-*O*-*β*-D-glucoside **(6)**

[Figure S22.^1^H-NMR (400 MHz, *d6*-DMSO) spectrum of](#_Toc476257005) 6-methyl-7-acetyl-1, 8-dihydroxy naphthalene-1-*O*-*β*-D-glucoside **(7)**

[Figure S23. APT spectrum of 6-](#_Toc476257006)methyl-7-acetyl-1, 8-dihydroxy naphthalene-1-*O*-*β*-D-glucoside **(7)**

Figure S24. HRESIMS^-^ spectrum of 6-methyl-7-acetyl-1, 8-dihydroxy naphthalene-1-*O*-*β*-D-glucoside **(7)**

[Figure S25. ^1^H NMR spectrum (400 MHz, MeOD) of](#_Toc476257024) ethyl β-D-glucopyranoside **(8)**

[Figure S26. APT spectrum of](#_Toc476257018) ethyl β-D-glucopyranoside **(8)**

Figure S27. HMBC spectrum of ethyl β-D-glucopyranoside **(8)**

Figure S28: Extraction and Isolation Flowchart.

Table S1. The concentration of total phenolic compounds and oxalic acid values of different extract/fractions prepared from roots of *R. vesicarius*


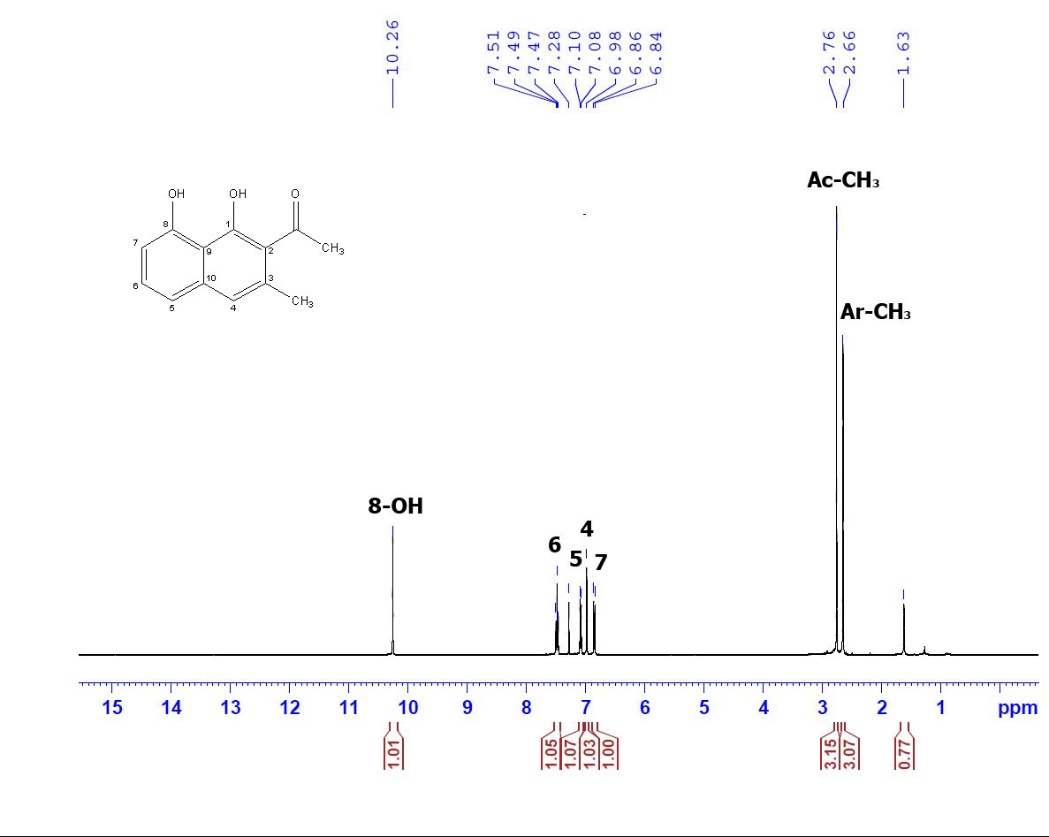


Figure S1. ^1^H-NMR spectrum (400 MHz, CDCL_3_) of Nepodin (**1)**


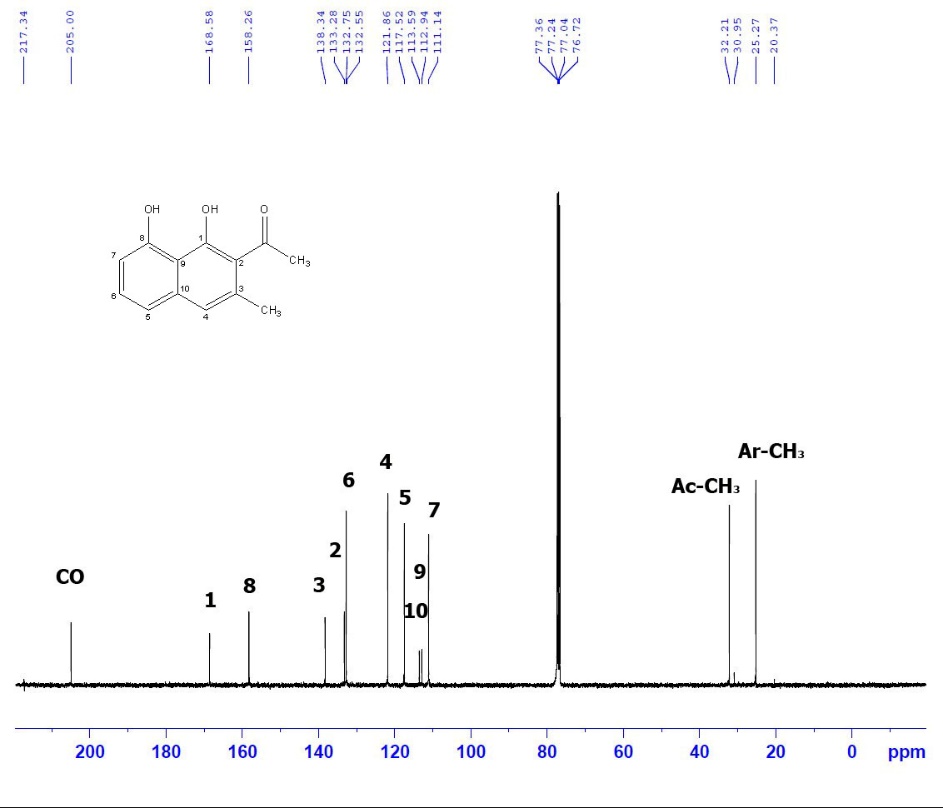


[Figure S2. ^13^C-NMR spectrum of](#_Toc476257013) Nepodin (**1)**


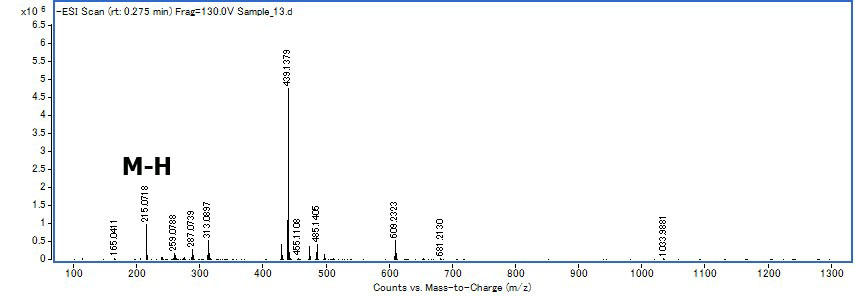


Figure S3. HRESIMS^-^ spectrum of Nepodin (**1)**


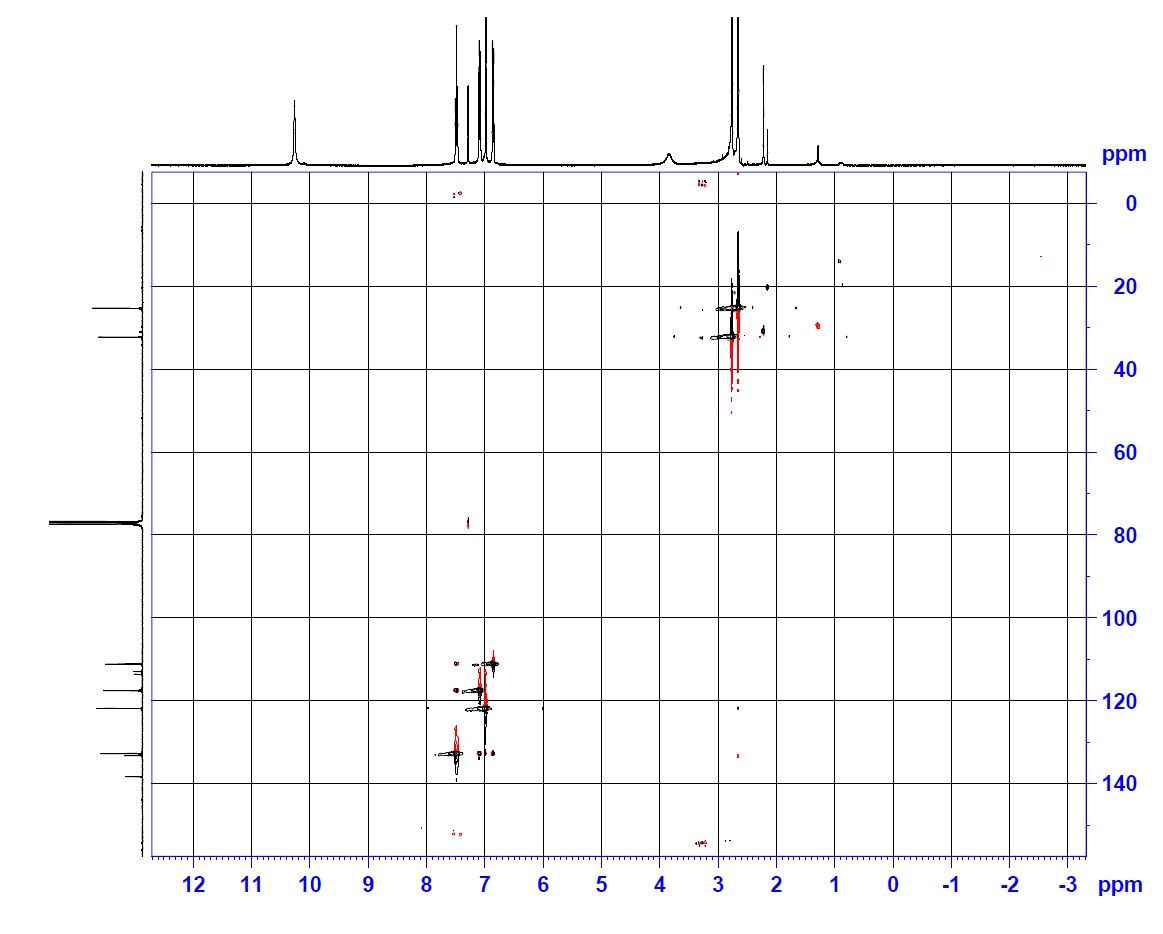


Figure S4. HSQC spectrum of Nepodin (**1)**


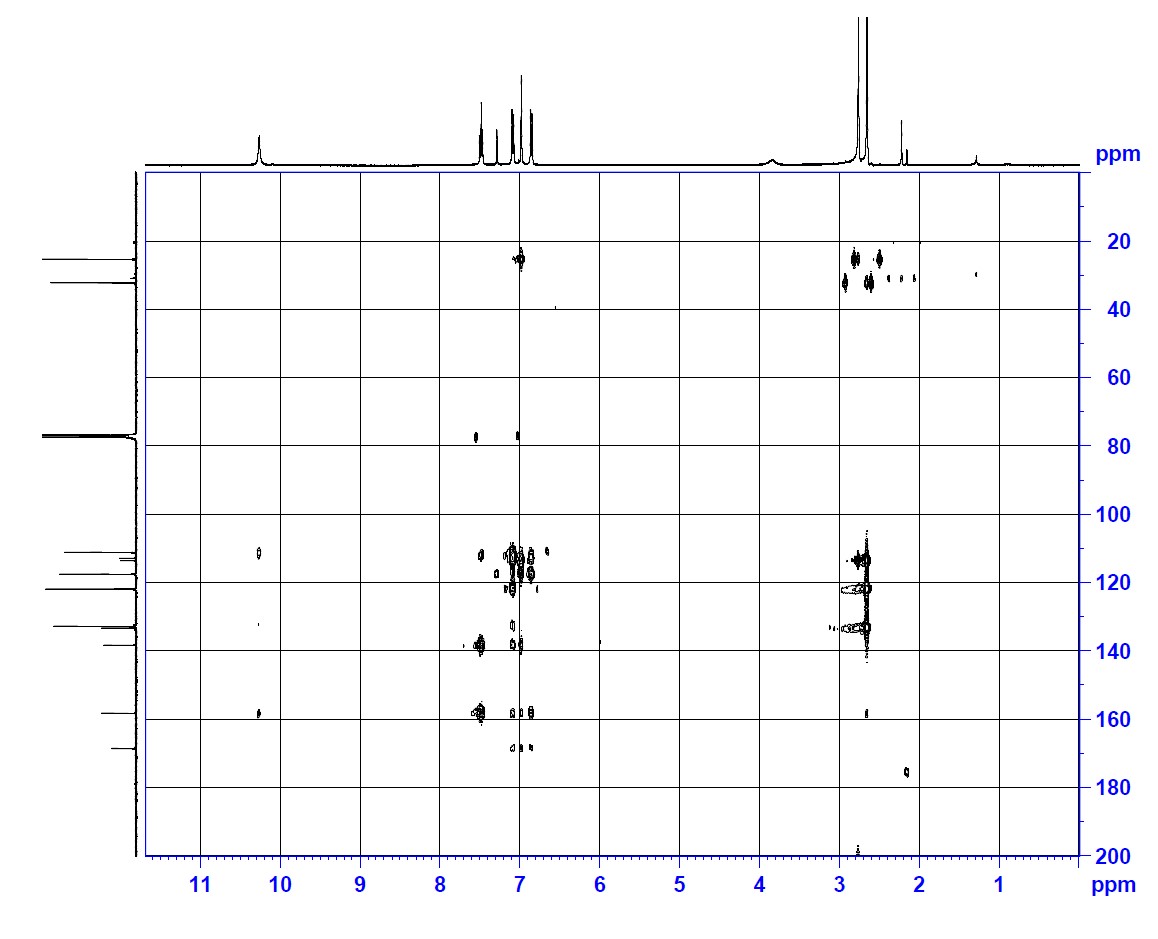


Figure S5. HMBC spectrum of Nepodin (**1)**


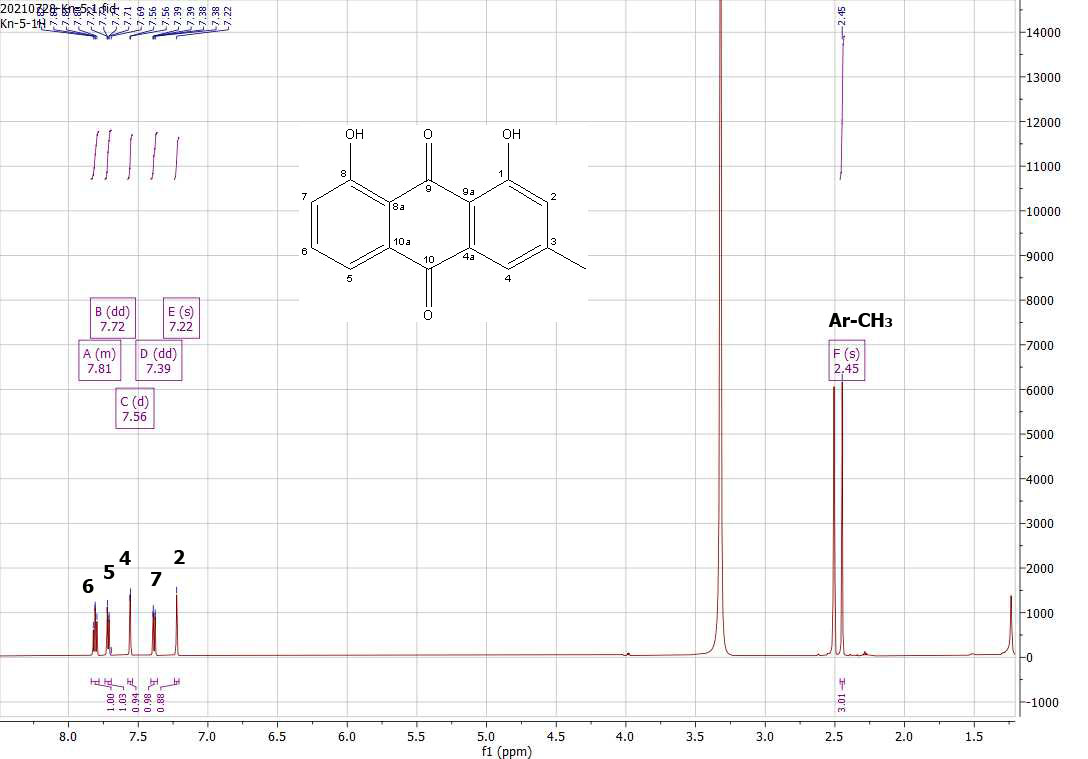


[Figure S6. ^1^H-NMR spectrum (400 MHz, CDCl_3_) of chrysophanol **(2 -**](#_Toc476257017)**)**


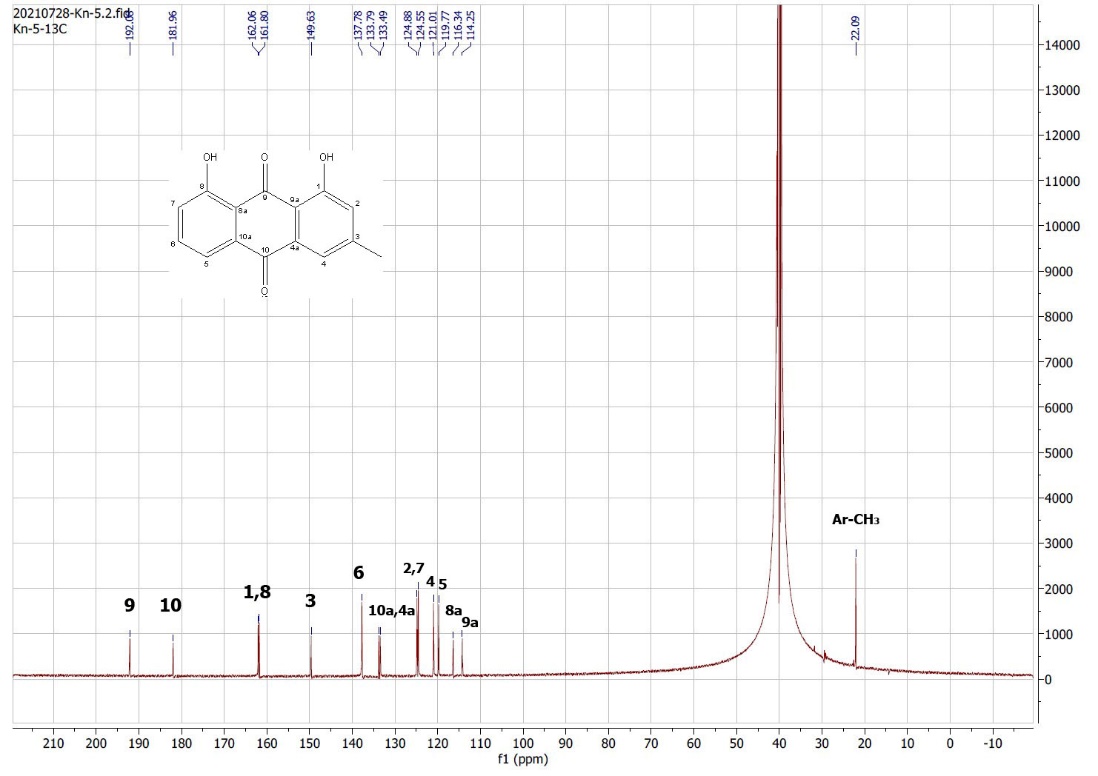


[Figure S7. ^13^C-NMR spectrum of chrysophanol **(2)**](#_Toc476257018)


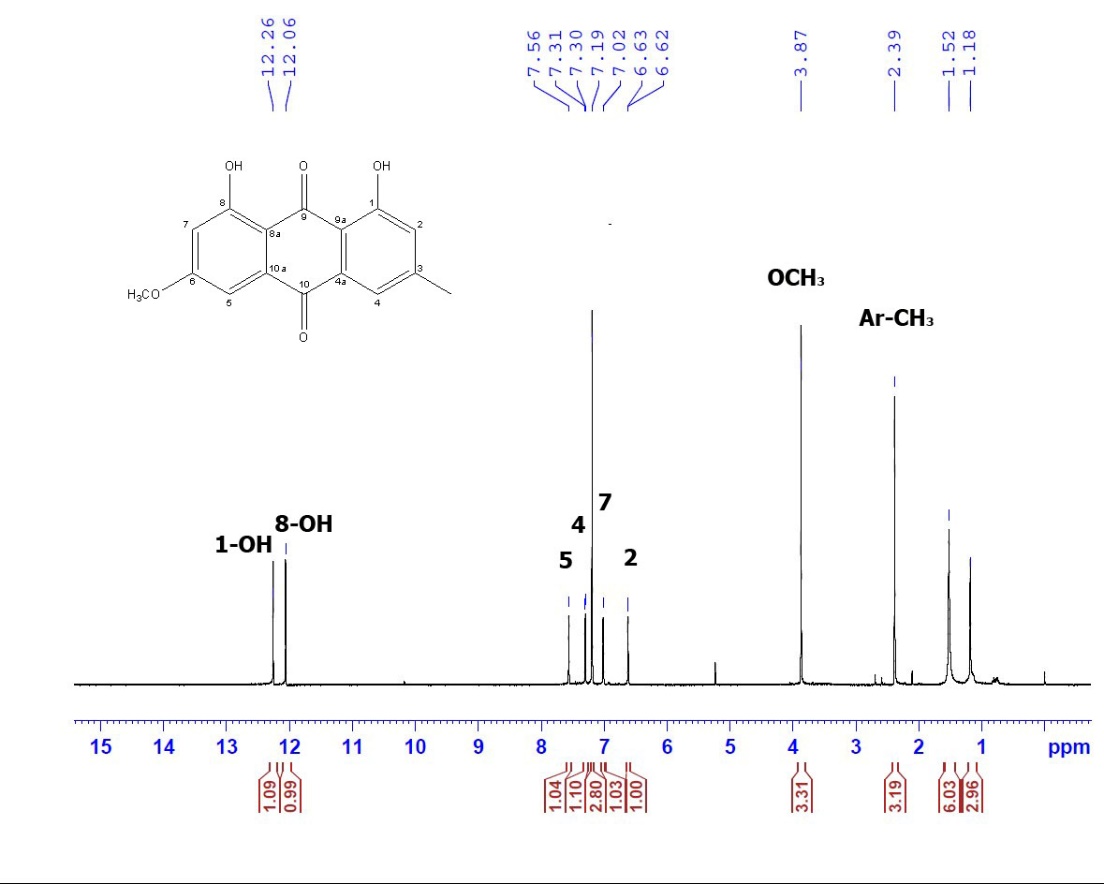


Figure S8. 1H-NMR (400 MHz, CDCl3) spectrum of physcion (3)


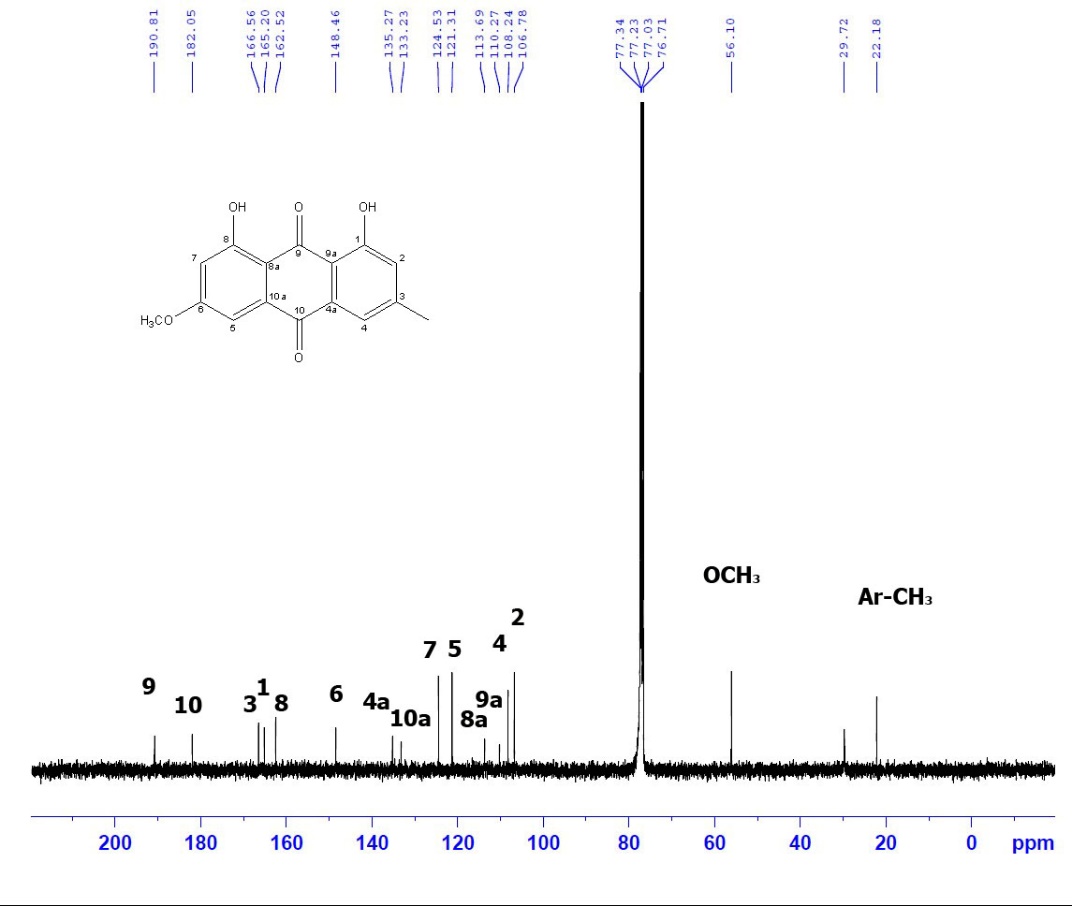


[Figure S9. ^13^C-NMR spectrum of physcion **(3)**](#_Toc476257006)


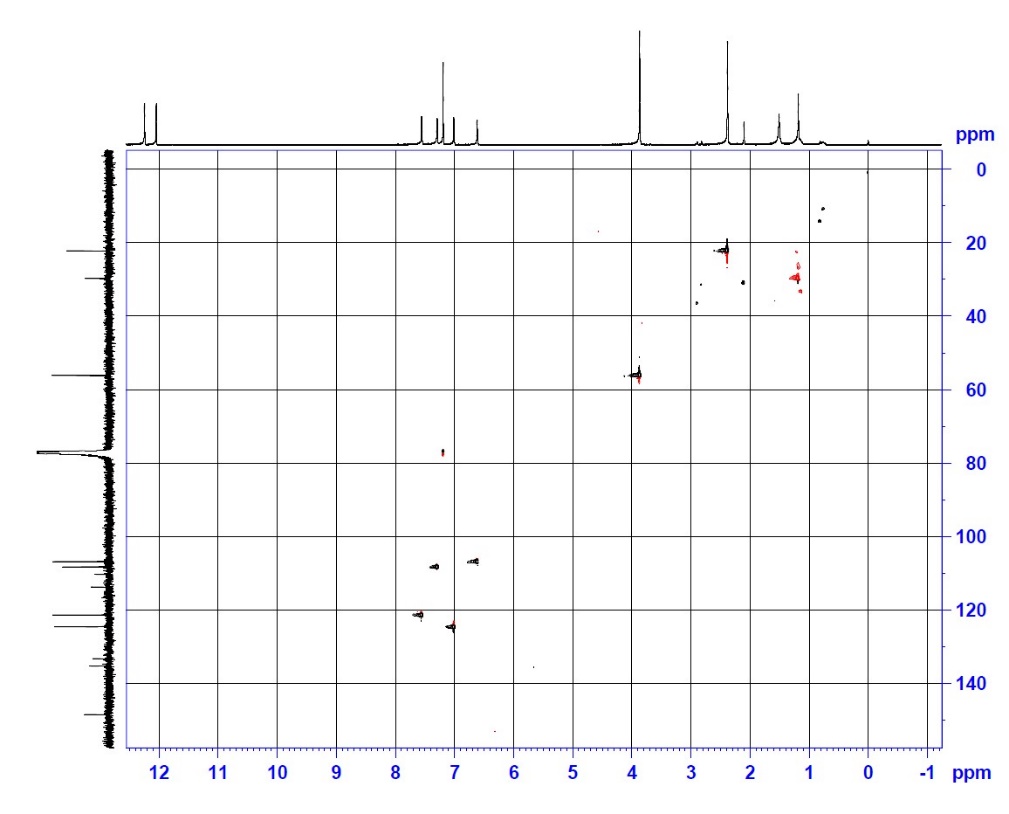


Figure S10. HSQC spectrum of physcion **(3)**


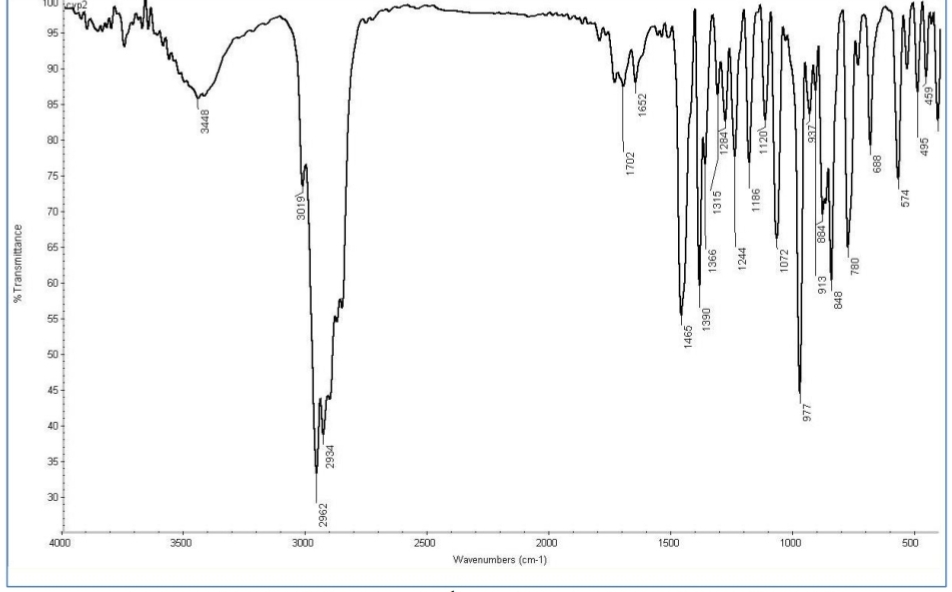


Figure S11. IR spectrum of *β-*sitosterol **(4)**


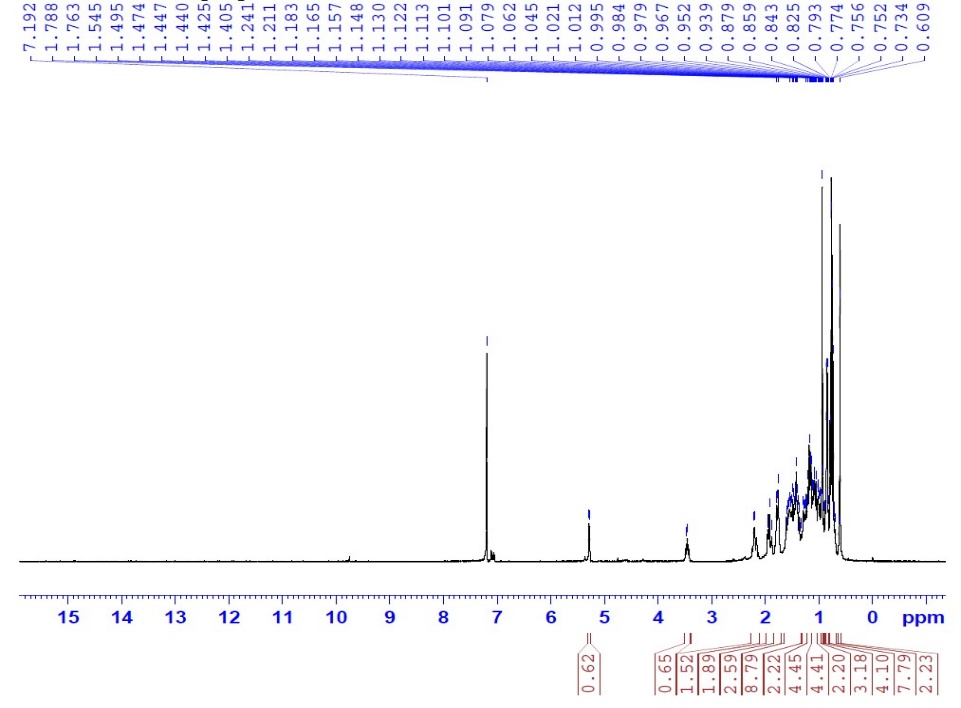


[Figure S12. ^1^H-NMR spectrum (400 MHz, CDCl_3_) of *β*-sitosterol (**4(**](#_Toc476257024)


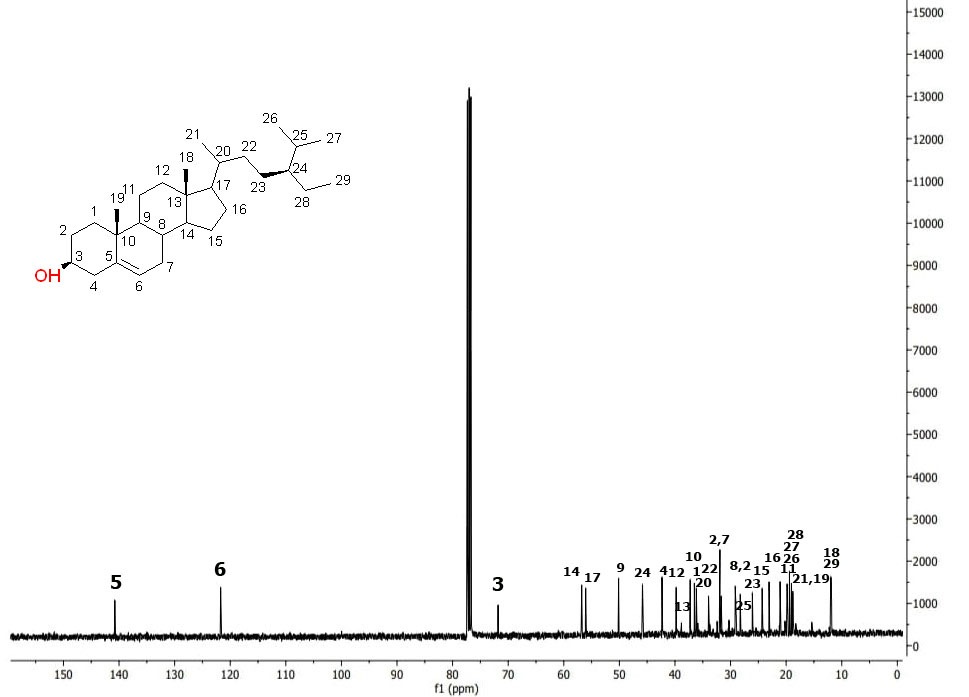


[Figure S13. ^13^C-NMR spectrum of *β*-sitosterol (**4(**](#_Toc476257024)


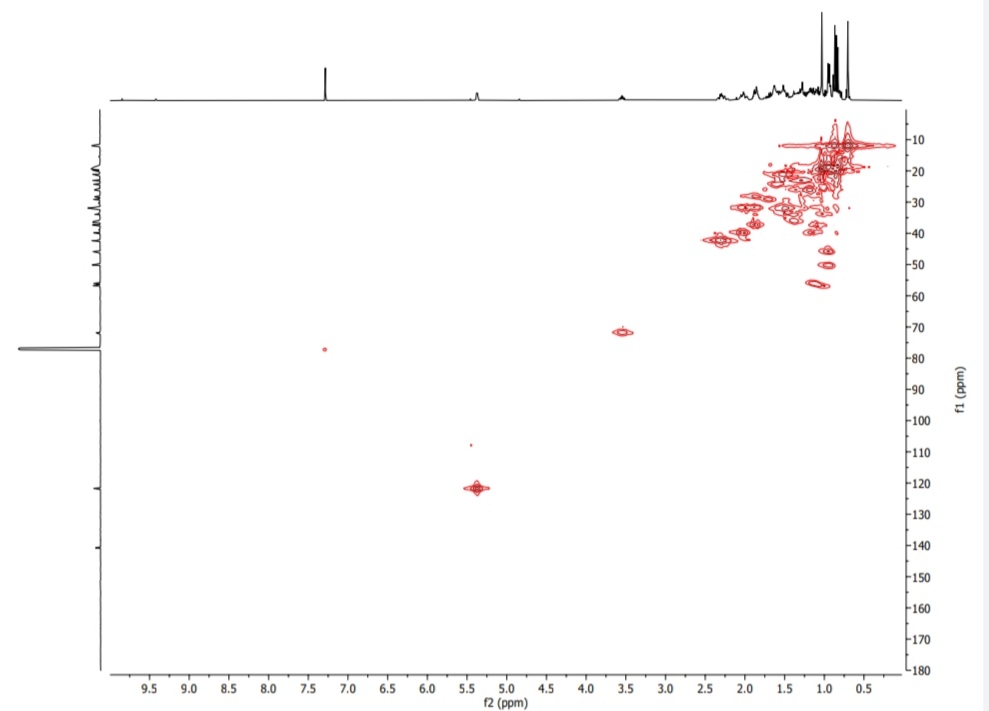


[Figure S14. HSQC spectrum of *β*-sitosterol (**4(**](#_Toc476257024)


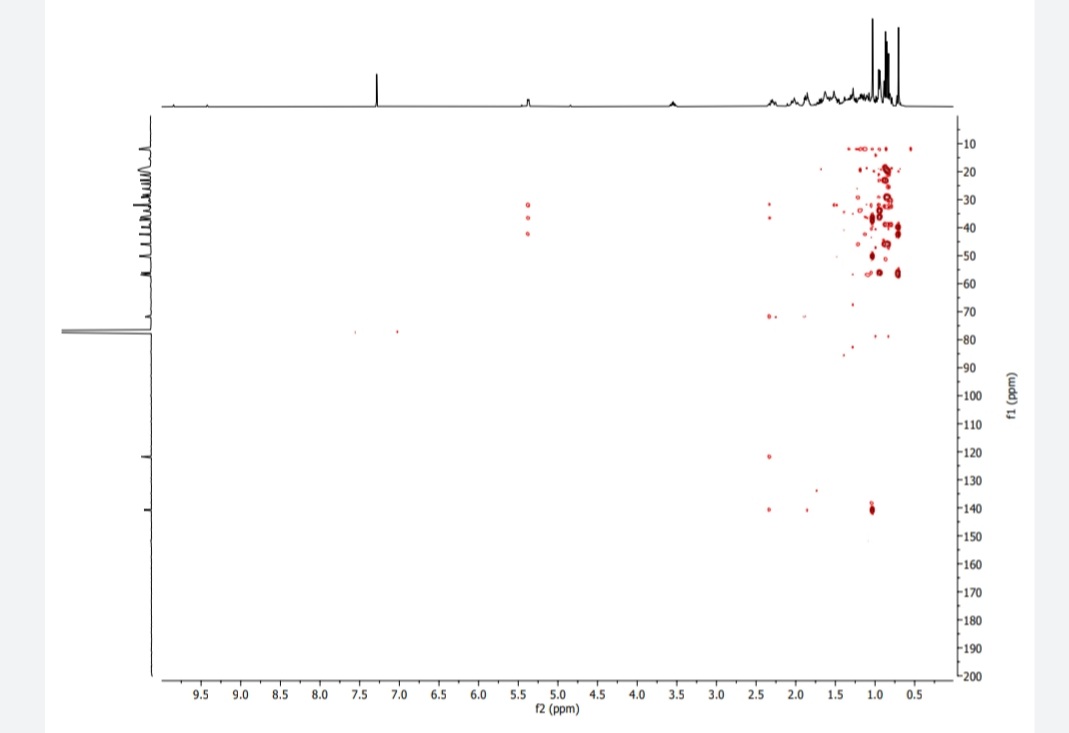


[Figure S15. HMBC spectrum of *β*-sitosterol (**4(**](#_Toc476257024)


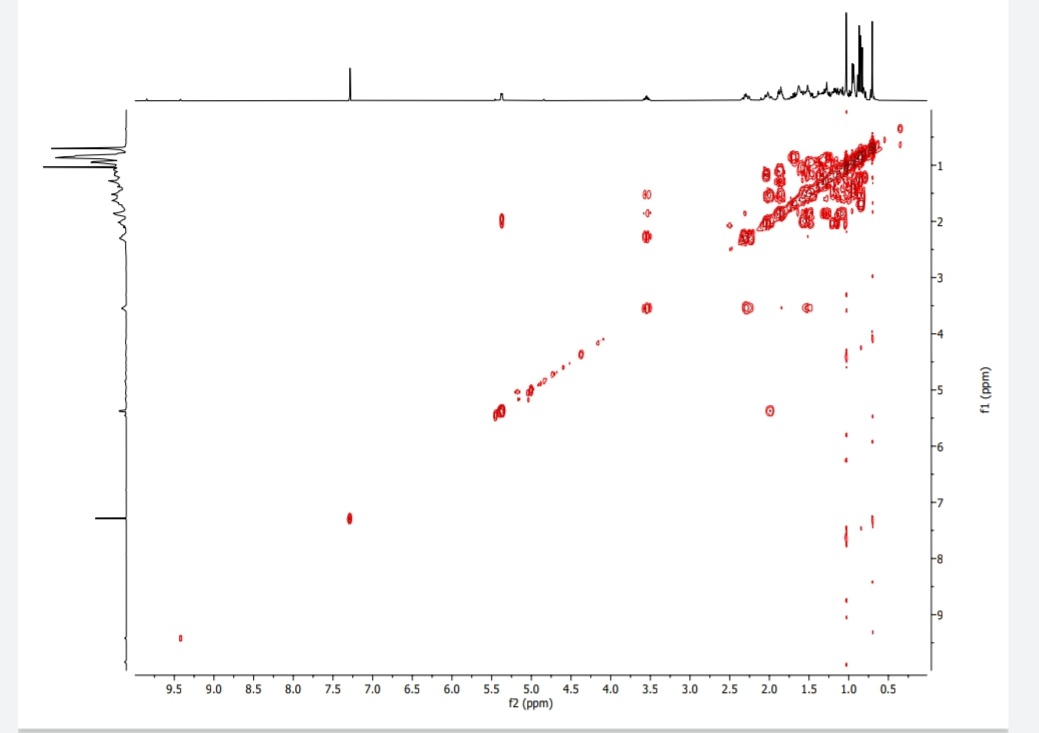


[Figure S16. COSY spectrum of *β*-sitosterol (**4(**](#_Toc476257024)


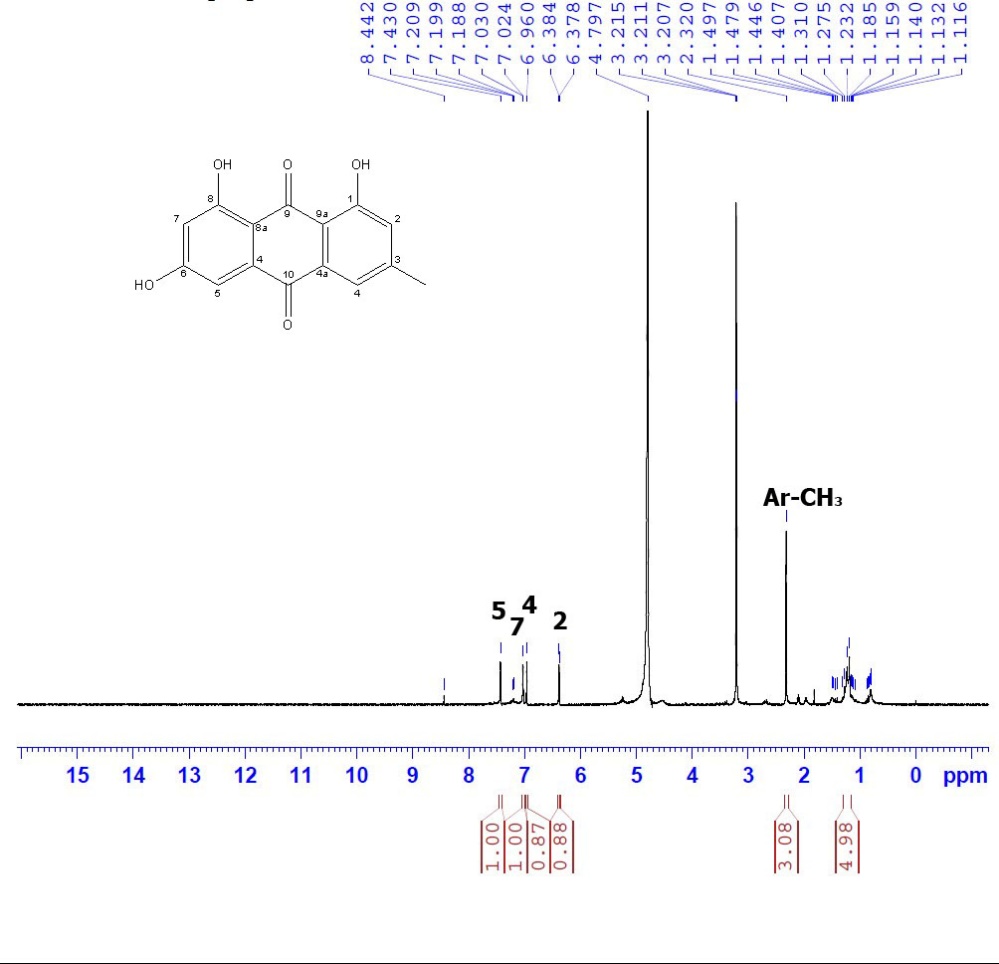


Figure S17. ^1^H-NMR spectrum (400 MHz, MeOD) of emodin (**5**)


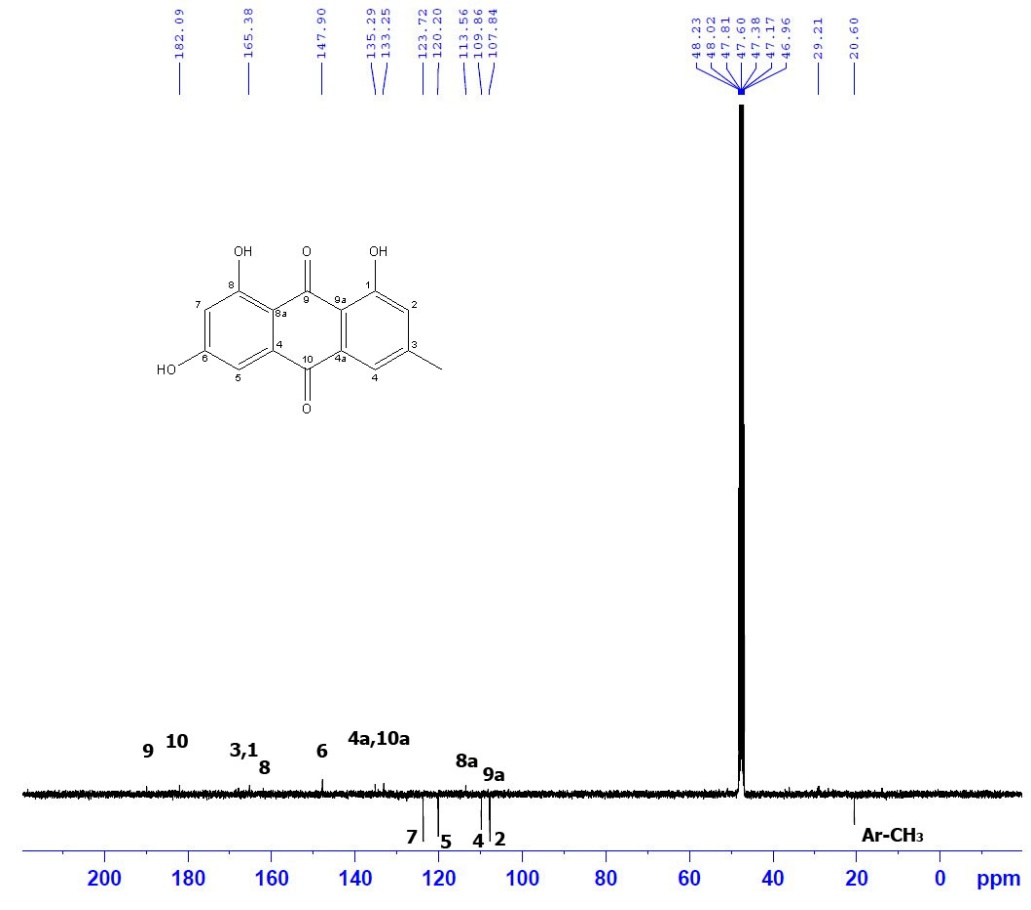


[Figure S18. APT spectrum of](#_Toc476257018) emodin **(5)**


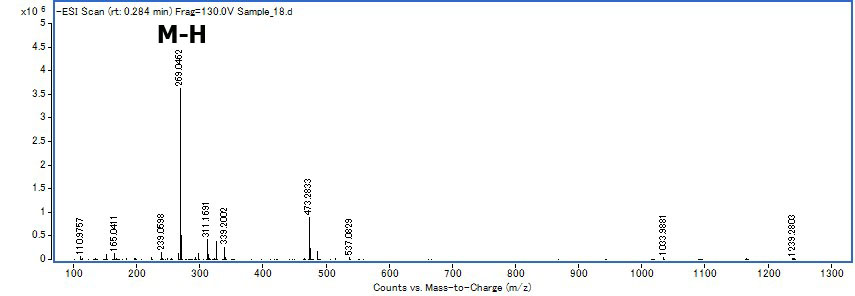


Figure S19. HRESIMS^-^ spectrum of emodin **(5)**


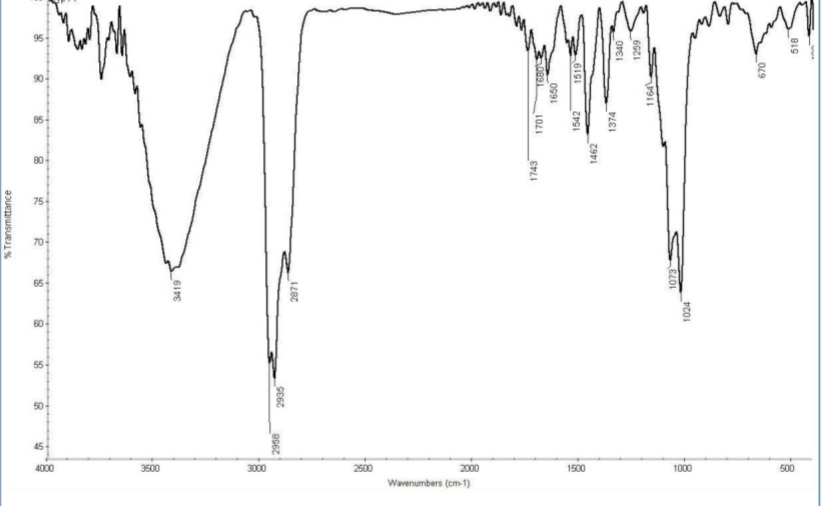


Figure S20. IR spectrum of *β*- sitosterol 3-*O*-*β*-D-glucoside **(6)**


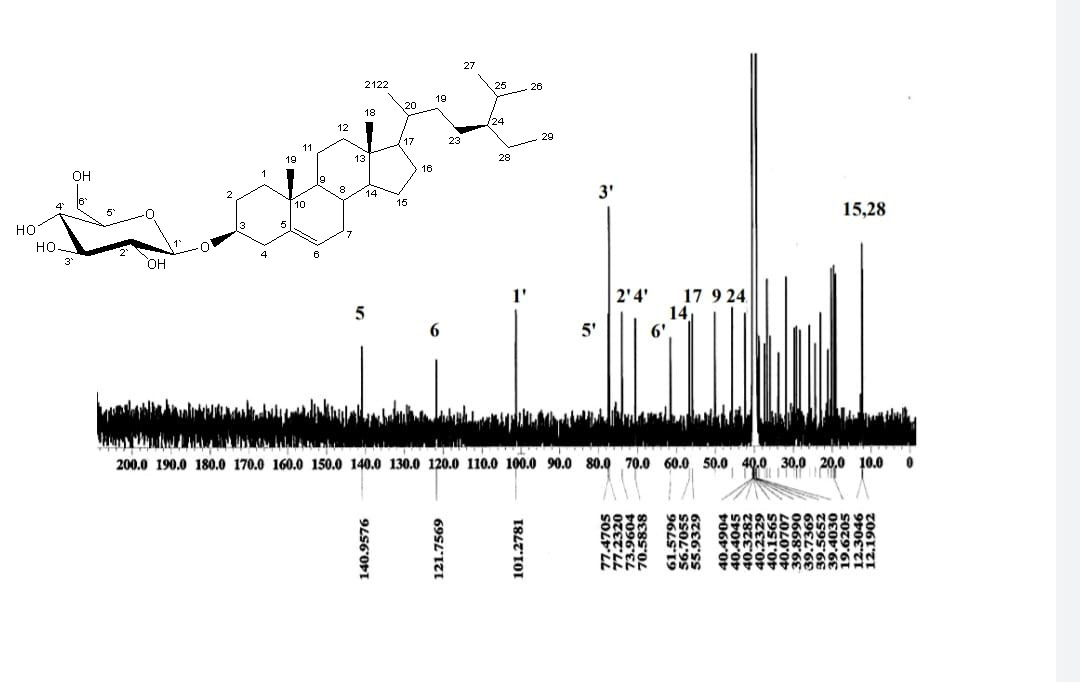
 Figure S21. ^13^C-NMR spectrum (400 MHz, d6-DMSO) of *β*- sitosterol 3-*O*-*β*-D-glucoside **(6)**


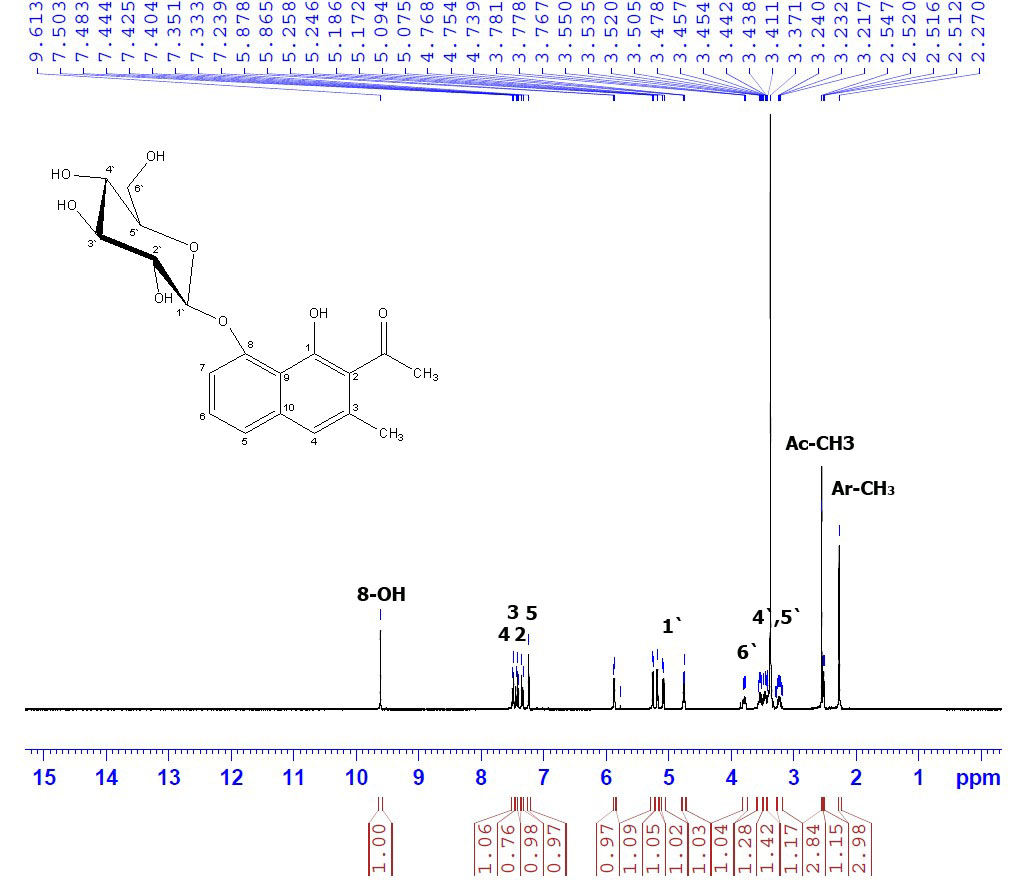


[Figure S22.^1^H-NMR (400 MHz, *d6*-DMSO) spectrum of](#_Toc476257005) 6-methyl-7-acetyl-1, 8-dihydroxy naphthalene-1-*O*-*β*-D-glucoside **(7)**


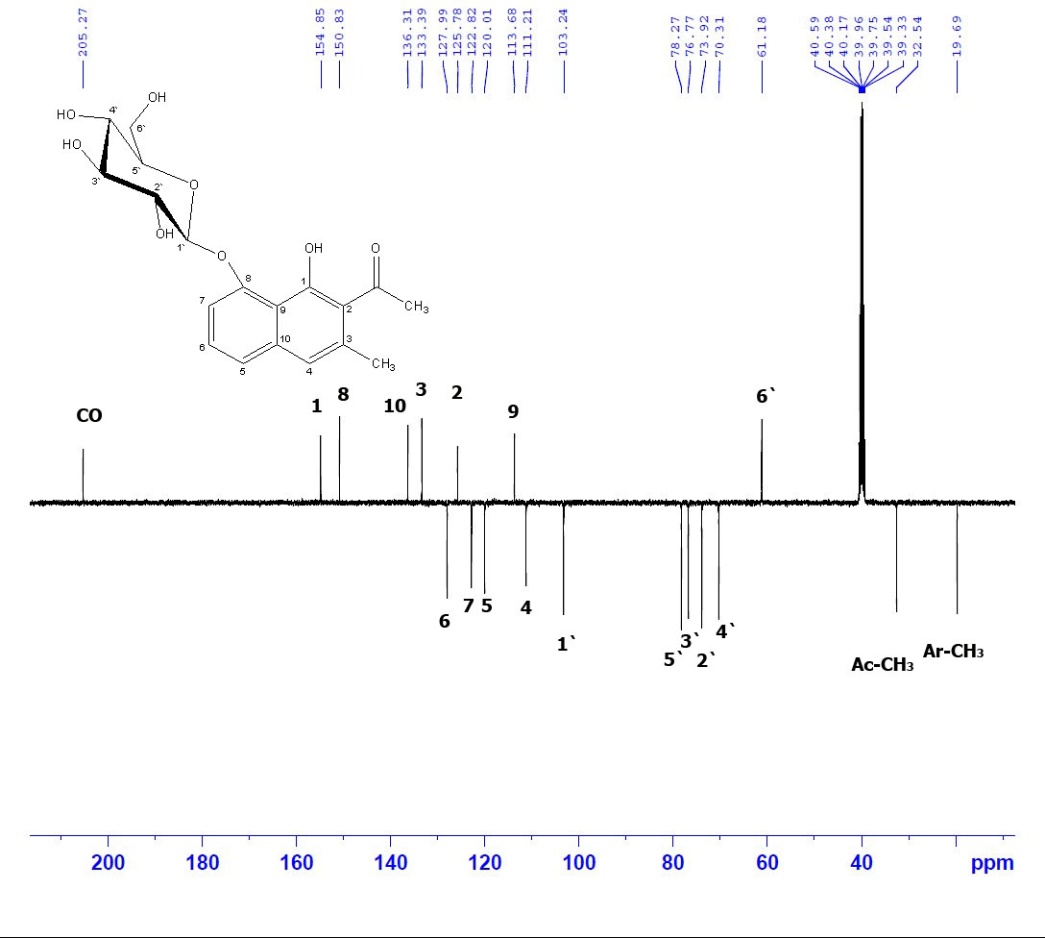


[Figure S23. APT spectrum of 6-](#_Toc476257006)methyl-7-acetyl-1, 8-dihydroxy naphthalene-1-*O*-*β*-D-glucoside **(7)**


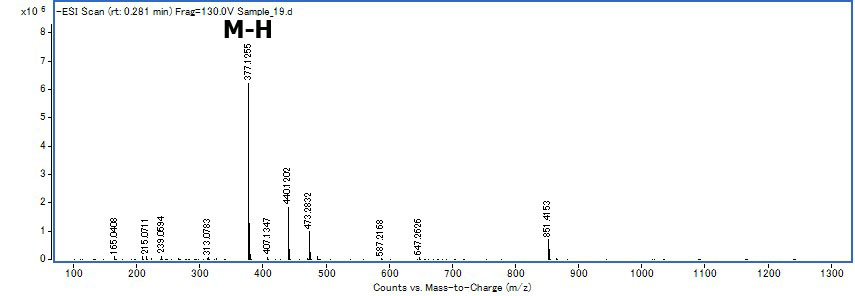


Figure S24. HRESIMS^-^ spectrum of 6-methyl-7-acetyl-1, 8-dihydroxy naphthalene-1-*O*-*β*-D-glucoside **(7)**


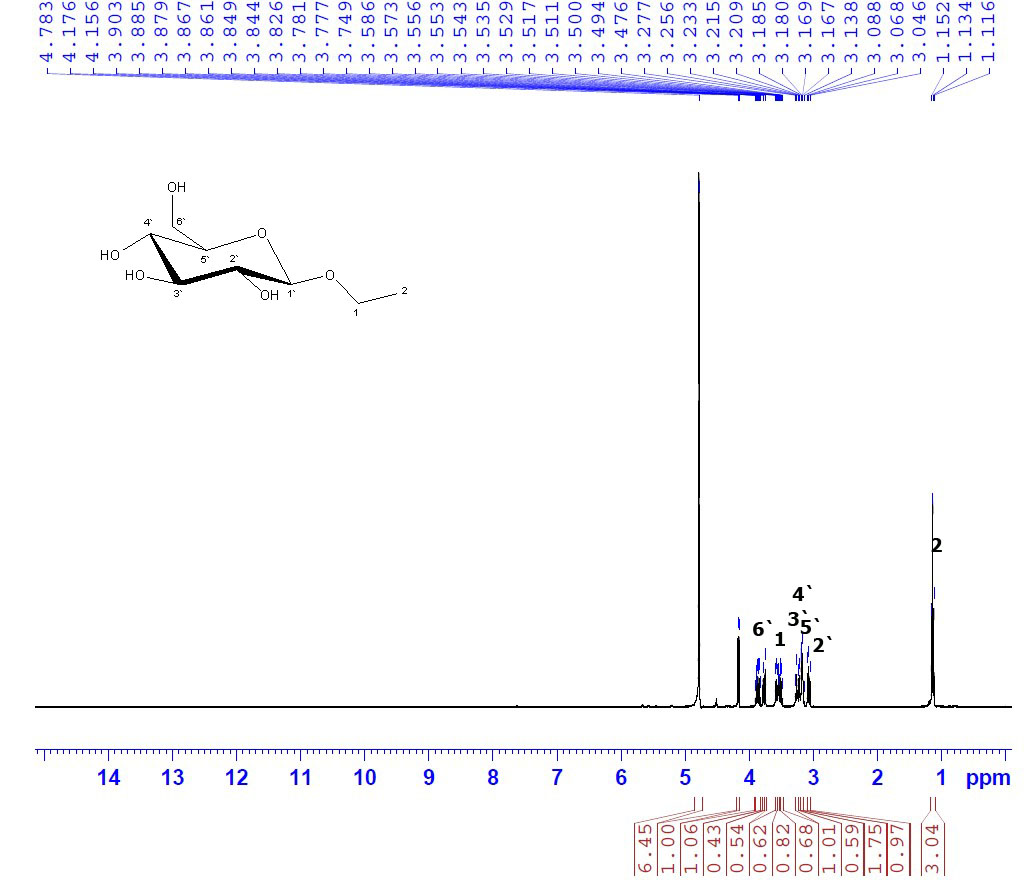


[Figure S25. ^1^H NMR spectrum (400 MHz, MeOD) of](#_Toc476257024) ethyl β-D-glucopyranoside **(8)**


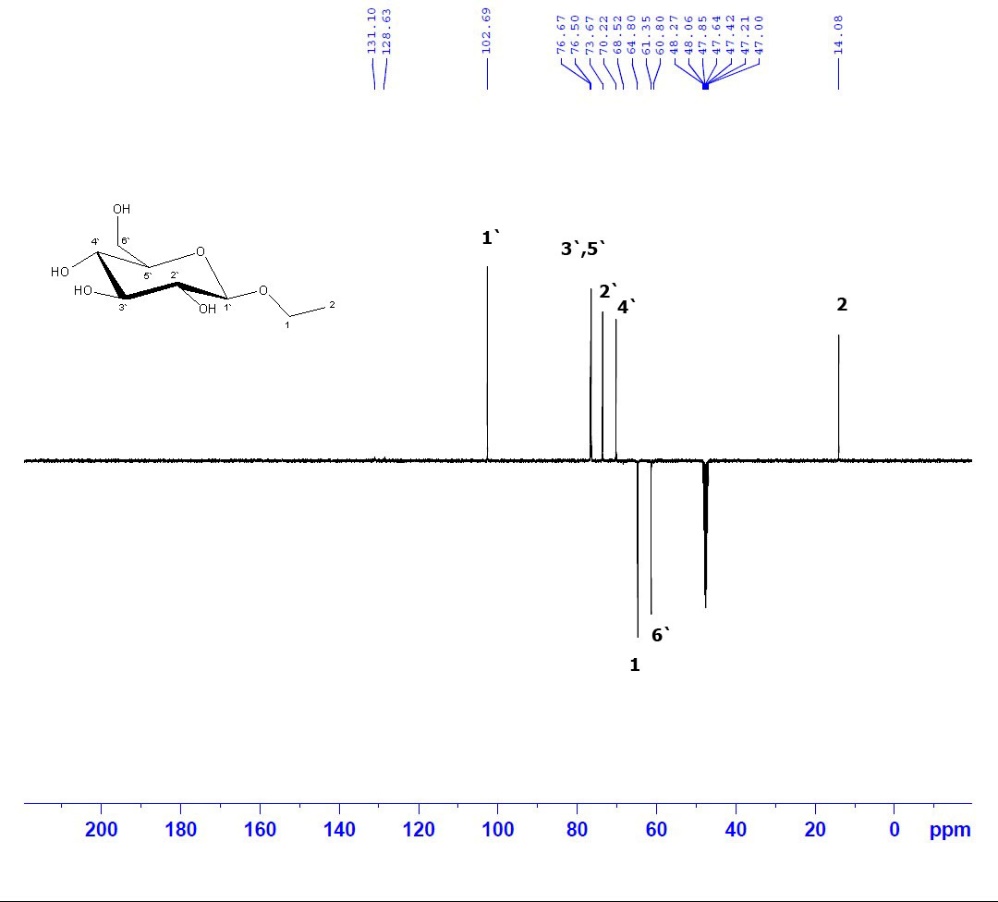


[Figure S26. APT spectrum of](#_Toc476257018) ethyl β-D-glucopyranoside **(8)**


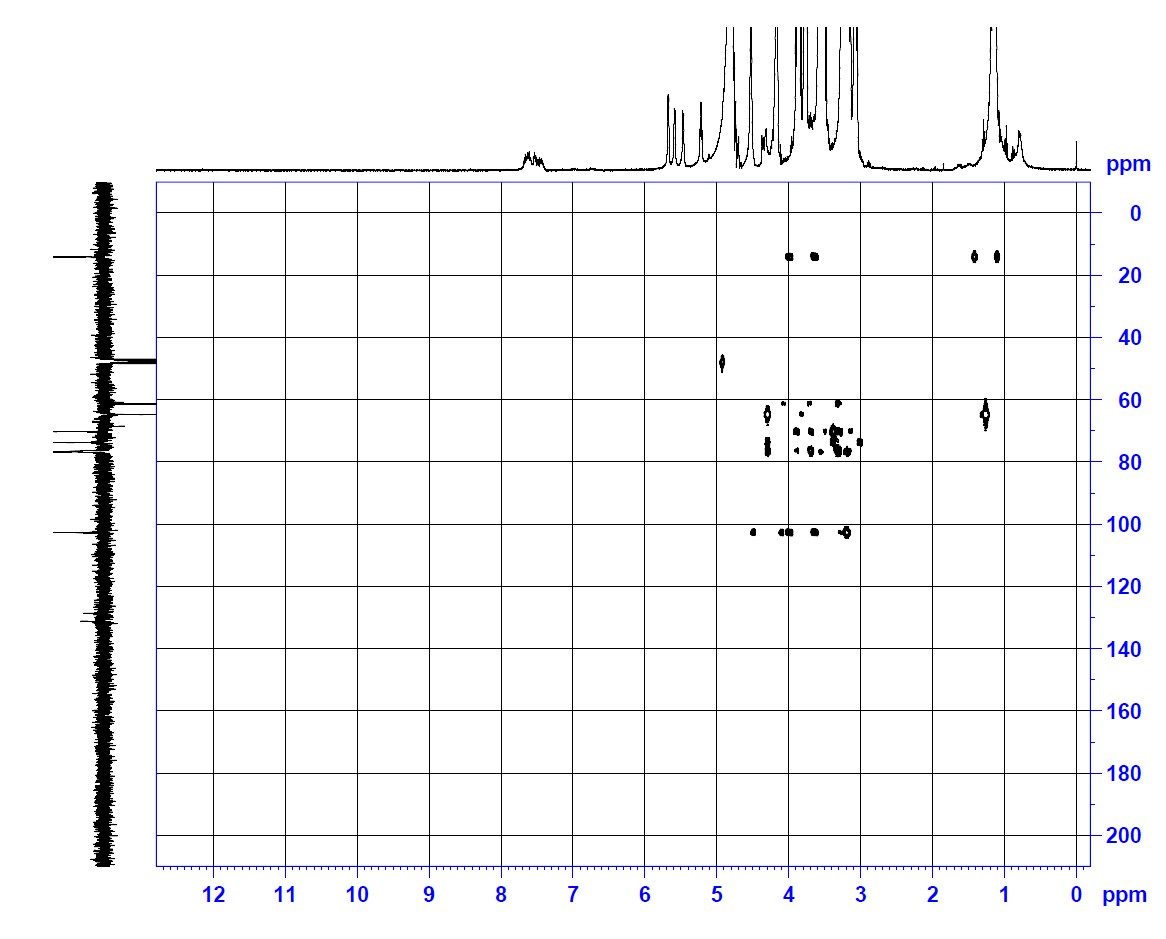


Figure S27. HMBC spectrum of ethyl β-D-glucopyranoside **(8)**

Figure S28: Extraction and Isolation Flowchart.

Table S1: The concentration of total phenolic compounds and oxalic acid values of different extract/fractions prepard from roots of *R. vesicarius*

| **Extract/ Fractions** | **Total phenolic contents (mM GAE/g)** | **Oxalic acid (mg/100 g)** |
| --- | --- | --- |
| **Total extract** | 68.07 ± 1.04 ^b^ | 4.37 ± 1.18^a^ |
| **Petroleum ether fraction** | 59.82 ± 0.87 ^c^ | 2.57 ± 0.67^c^ |
| **Ethyl acetate fractions** | 80.22 ± 1.2 ^a^ | 3.22 ± 1.24^b^ |

Values are means of three replications ± SD.

Different letters (lowercase) within the same column, means significantly different at *P* ˂ 0.05.
